# Cholinergic signaling via the α7 nicotinic acetylcholine receptor regulates the migration of monocyte-derived macrophages during acute inflammation

**DOI:** 10.1186/s12974-023-03001-7

**Published:** 2024-01-04

**Authors:** Kasey R. Keever, Kui Cui, Jared L. Casteel, Sanjay Singh, Donald B. Hoover, David L. Williams, Valentin A. Pavlov, Valentin P. Yakubenko

**Affiliations:** 1 Department of Biomedical Sciences, Quillen College of Medicine, East Tennessee State University, PO Box 70582, Johnson, TN USA; 2 Department of Surgery, Quillen College of Medicine, East Tennessee State University, Johnson, TN USA; 3 Center of Excellence in Inflammation, Infectious Disease and Immunity, East Tennessee State University, Johnson, TN USA; 4 Center for Biomedical Science and Center for Bioelectronic Medicine, The Feinstein Institute for Medical Research, Northwell Health, Manhasset, NY USA; 5 Donald and Barbara Zucker School of Medicine at Hofstra/Northwell, Hempstead, NY 11550 USA

**Keywords:** Cholinergic anti-inflammatory pathway, α7nAChR, Macrophage, Migration, Endotoxemia, Sepsis

## Abstract

**Background:**

The involvement of the autonomic nervous system in the regulation of inflammation is an emerging concept with significant potential for clinical applications. Recent studies demonstrate that stimulating the vagus nerve activates the cholinergic anti-inflammatory pathway that inhibits pro-inflammatory cytokines and controls inflammation. The α7 nicotinic acetylcholine receptor (α7nAChR) on macrophages plays a key role in mediating cholinergic anti-inflammatory effects through a downstream intracellular mechanism involving inhibition of NF-κB signaling, which results in suppression of pro-inflammatory cytokine production. However, the role of the α7nAChR in the regulation of other aspects of the immune response, including the recruitment of monocytes/macrophages to the site of inflammation remained poorly understood.

**Results:**

We observed an increased mortality in α7nAChR-deficient mice (compared with wild-type controls) in mice with endotoxemia, which was paralleled with a significant reduction in the number of monocyte-derived macrophages in the lungs. Corroborating these results, fluorescently labeled α7nAChR-deficient monocytes adoptively transferred to WT mice showed significantly diminished recruitment to the inflamed tissue. α7nAChR deficiency did not affect monocyte 2D transmigration across an endothelial monolayer, but it significantly decreased the migration of macrophages in a 3D fibrin matrix. In vitro analysis of major adhesive receptors (L-selectin, β1 and β2 integrins) and chemokine receptors (CCR2 and CCR5) revealed reduced expression of integrin αM and αX on α7nAChR-deficient macrophages. Decreased expression of αMβ2 was confirmed on fluorescently labeled, adoptively transferred α7nAChR-deficient macrophages in the lungs of endotoxemic mice, indicating a potential mechanism for α7nAChR-mediated migration.

**Conclusions:**

We demonstrate a novel role for the α7nAChR in mediating macrophage recruitment to inflamed tissue, which indicates an important new aspect of the cholinergic regulation of immune responses and inflammation.

**Supplementary Information:**

The online version contains supplementary material available at 10.1186/s12974-023-03001-7.

## Background

Active research during the last 20 years has revealed the important role of the vagus nerve in the regulation of immunity and inflammation in a physiological mechanism termed *the inflammatory reflex* [1, 2]. In the inflammatory reflex, sensory (afferent) vagus nerve signaling is activated by cytokines and other inflammatory molecules in response to pathogens, injury, or other pathophysiological events [1, 3]. This signaling is integrated in the brainstem with motor (efferent) vagus nerve cholinergic signaling, which controls pro-inflammatory cytokine levels and inflammation [2, 4]. This efferent arm of the inflammatory reflex was termed *the cholinergic anti-inflammatory pathway* [2, 5]. The α7 nicotinic acetylcholine receptor (α7nAChR) expressed on macrophages and other immune cells has been identified as a key mediator of cholinergic anti-inflammatory signaling [6, 7]. Stimulation of the α7nAChR on macrophages activates downstream intracellular mechanisms, including suppression of NF-κB activation and results in decreased production of TNF and other pro-inflammatory cytokines [8–11]. These discoveries opened an avenue of preclinical research revealing the anti-inflammatory efficacy of vagus nerve stimulation (VNS) and α7nAChR agonists in endotoxemia, sepsis and many other inflammatory conditions [12, 13]. This research paved the way to recent successful clinical trials with VNS in patients with inflammatory disorders [14].

Murine endotoxemia and cecal ligation and puncture (CLP) have been widely used in studying the role of the α7nAChR in the cholinergic regulation of inflammation. Endotoxemia, associated with robust systemic cytokine release and inflammation is considered by some as a model of gram negative sepsis, while CLP is a clinically relevant model of polymicrobial sepsis [15]. Sepsis is a life-threatening condition characterized by organ dysfunction resulting from an excessive inflammatory response to infection. This organ system dysfunction is correlated with higher long term mortality, even if patients recover from their illness in the hospital [16]. In mice, VNS or pharmacological cholinergic α7nAChR activation suppresses pro-inflammatory cytokine levels and mitigate mortality in mice with endotoxemia and CLP [13, 17–20]. The role of macrophages in endotoxemia and sepsis is complex; some reports characterize macrophages as protective due to their crucial role in efferocytosis of neutrophils, phagocytosis of bacteria, and tissue repair, while other reports indicate their detrimental effects [21–23]. The role of α7nAChR on macrophages in mediating cholinergic suppression of pro-inflammatory cytokine production in the cholinergic anti-inflammatory pathway has been characterized as a major mechanism underlying the neural control of immune responses. However, other potential mechanisms, such as modulating the recruitment of monocytes/macrophages to damaged tissue, remain unclear.

In this study, we investigated the broader role of the α7nAChR in inflammation by examining the migration and accumulation of macrophages during endotoxemia. We showed that the protective role of α7nAChR in endotoxemia is positively correlated with monocyte/macrophage migration to the inflamed tissues. Moreover, we found that α7nAChR-mediated migratory properties depend on the expression of a major adhesive receptor integrin αMβ2 (CD11b/CD18), thus indicating an important molecular link between cholinergic signaling and macrophage motility. Therefore, these results reveal a novel protective mechanism of the cholinergic anti-inflammatory pathway.

## Materials and methods

### Reagents and antibodies

Reagents were purchased from Sigma-Aldrich (St. Louis, MO, USA), BioRad (Hercules, CA, USA), BioLegend (San Diego, CA, USA), and Thermo Fisher Scientific (Waltham, MA, USA). Lipopolysaccharide (LPS, endotoxin) derived from *E. coli* O55:B5, and PNU-282987 were purchased through Sigma-Aldrich. Antibodies against cell surface markers Ly6-G (clone 1AB), Ly6-C (clone HK1.4), αM (clone M1/70), αL (clone M17/4), F4/80 (clone BM8), αX (clone N418), and α4 (clone R1–2) are from eBioscience. Antibodies against Siglec F (clone 1RNM44N) and L-selectin (clone MEL-14) are from Invitrogen. Antibodies against CCR2 (clone SA203G11) and CCR5 (clone HM-CCR5) are from BioLegend.

### Animals

Wild-type (WT; C57BL/6J, stock #000664) and α7nAChR-deficient (α7nAChR^−/−^; B6.129S7-Chrna7^tm1Bay^/J, stock #003232) mouse colonies were purchased from Jackson Laboratory (Bar Harbor, ME, USA). The α7nAChR deficient strain was backcrossed to C57BL/6 for eight generations. Mice aged between 8 and 12 weeks were used for the study. Similar age WT and α7nAChR^−/−^ mice were employed for each experiment. No comparative analysis was conducted across mice of varying sexes and ages. All animal procedures were performed according to animal protocols approved by East Tennessee State University IACUC. Protocol number is P210903.

### Endotoxemia

In survival experiments, male or female WT and α7nAChR^−/−^ mice were intraperitoneally injected with a sublethal dose of LPS calculated based on body weight. Since LPS activity is slightly variable from bath to bath, we evaluated the sublethal dose for each vial preparation using increasing doses of LPS (*E. coli* O55:B5) in C57BL/6J wild-type mice. Female mice are more resistant to LPS treatment compared to male mice [24, 25]. Therefore, a gender-specific dosing was applied to reach a similar percentage of lethality for male and female mice. Depending on the LPS bath preparation we used 7–8 mg/kg for males and 9–12 mg/kg for females. Notably, the same concentration of LPS was used for all groups in each experiment.

In all endotoxemia experiments, body temperature was monitored twice daily using a rectal probe connected to a ThermoWorks (American Fork, UT, USA) MicroTherma meter.

To examine macrophage accumulation in the lungs, male or female WT and α7nAChR^−/−^ mice were given an intraperitoneal injection of LPS as described above. After 48h, mice were euthanized using Isothesia (Henry Schein Animal Health, Dublin, OH) and perfused, and lungs were removed. Lungs were digested using collagenase II as described below ( "Flow Cytometry and Imaging Flow Cytometry Analyses" section) and prepared for flow cytometry. In an additional experiment, male and female WT mice were treated intraperitoneally with 3mg/kg PNU-282987, 15 min before the injection of LPS, to examine the effect of α7nAChR stimulation on macrophage accumulation. Control mice received DMSO (vehicle) 15 min before LPS. Samples were incubated with anti-αM/PE-Cy7, anti-CCR2/APC, anti-CCR5/PE-Cy7, anti-Siglec F/FITC, anti-Ly6-G/PE, anti-F4/80/PE, anti-F4/80/APC, and anti-αX/APC across multiple samples.

### Isolation of peritoneal macrophages

Thioglycollate-induced peritoneal macrophages are a well-established source to study macrophage function in inflammatory conditions. Peritoneal macrophages from 8- to 12-week-old WT and α7nAChR^−/−^ mice were collected via peritoneal lavage with 5mL of sterile PBS 72 h after intraperitoneal injection of 1mL 4% thioglycolate. Mice were euthanized via CO_2_ asphyxiation before the collection procedure. The cells were counted and plated in petri dishes for 2 h in RPMI 1640 (Corning, Corning, NY) with 10% FBS and 1% penicillin/streptomycin**,** after which non-adherent cells were removed.

### Isolation of monocytes from mouse bone marrow

The isolation of bone-marrow-derived monocytes provides a pure population of monocytes that exceed the number of monocytes obtained from the peripheral blood of mice by approximately 10 folds. Monocytes were isolated from the femoral and tibial bone marrow of WT and α7nAChR^−/−^ mice by first flushing out bone marrow with RPMI 1640, followed by lysis of red blood cells. Magnetic-assisted cell sorting (MACS) was then used to purify monocytes via a negative separation kit, following the manufacturer’s protocol (Miltenyi Biotec, Gaithersburg, MD, USA). Purity of the isolated monocytes was analyzed by flow cytometry using antibodies to αM/PE-Cy7, Ly6-G/PE, and Ly6-C/FITC. In all experiments, the purity was between 87% and 92%.

### Adoptive transfer of monocytes in the model of endotoxemia

Monocytes were isolated from the bone marrow of WT and α7nAChR^−/−^ mice, as above, and labeled with red PKH26 (WT), or green PKH67 (α7nAChR^−/−^) fluorescent dyes. A total of 1X10^6^ red and 1X10^6^ green monocytes were mixed equally and injected into the tail veins of WT mice or α7nAChR^−/−^ mice. These mice received a sub-lethal dose of LPS intraperitoneally within 5 min after injection of cells. After 48 h, the mice were sacrificed using Isothesia and perfused with PBS. Lungs, liver, and spleen were isolated and digested with 2mg/mL collagenase II (Sigma Aldrich, St Louis, MO, USA) prepared in HBSS as previously described [26]. Digested cell suspension was filtered through a 70μm cell strainer and any remaining red blood cells were lysed. Cell filtrate was incubated with a viability dye and analyzed using flow cytometry (Fortessa X-20, Becton Dickson, Franklin Lakes, NJ, USA) and imaging flow cytometry (ImageStream Mark II, Amnis, Seattle, WA, USA) for the detection of fluorescently labeled cells. The dye colors were used with only WT cells in a separate experiment to confirm that dye color does not influence the result.

### Adoptive transfer rescue in the model of endotoxemia

WT or α7nAChR^−/−^ monocytes were isolated from bone marrow as described above in  "Isolation of Monocytes from Mouse Bone Marrow" section. Freshly isolated cells, either WT or α7nAChR^−/−^, were injected into the tail veins of WT or α7nAChR^−/−^ recipient mice. Cell injection was immediately followed by a sub-lethal dose of LPS. In all adoptive transfer rescue experiments, body temperature and morbidity were monitored twice daily. Mortality rate was analyzed using the Kaplan–Meier method.

### Flow cytometry and imaging flow cytometry analyses

Flow cytometry analysis was used to assess cell surface markers listed in  "Reagents and Antibodies" section as well as determine the number of PHK26 and PKH67 positive cells in the lungs, liver, and spleen during adoptive transfer. For the analysis of cell surface markers, harvested cells were first incubated with FcR-Blocking solution (eBioscience) for 15 min on ice. Next, samples of 2 × 10^6^ cells were incubated with appropriate antibody panels for 30 min on ice. Cells were then washed and analyzed using the Fortessa X-20 (Becton Dickson).

To detect labeled macrophages in tissue, the lungs, liver, and spleen were digested using 2mg/mL collagenase II (Sigma-Aldrich, St Louis, MO, USA) as described above in "Adoptive Transfer of Monocytes in the Model of Endotoxemia" section. Cell suspension was next pre-cleaned via filtering through a 70μm cell strainer. Cells were incubated with live/dead viability dye for 30 min on ice (Thermo Fisher, Waltham, MA, USA). PKH26 and PKH67 labeled macrophages within the digested organs were analyzed with flow cytometry (Fortessa X-20) and imaging flow cytometry (Image Stream Mark II, Amnis). For analysis of αM on labeled macrophages, preparation was carried out as above.

Imaging flow cytometry analysis results were analyzed using IDEAS 6.2 software. The PKH26 and PKH67 labeled cells were captured on channels 2 and 3, respectively.

### Macrophage 3D migration assay

WT and α7nAChR^−/−^ peritoneal macrophages were labeled with PKH26 red fluorescent dye or PKH67 green fluorescent dye. An equal number of WT and α7nAChR^−/−^ were mixed and plated on the membranes of 6.5mm transwell inserts with 8μm pores (Costar, Corning, NY) pre-coated with 4μg/mL fibrinogen for 3 h. A 3-D fibrin gel was made by mixing 0.75mg/mL fibrinogen containing 1% FBS and 1% penicillin/streptomycin with 0.5 U/mL thrombin, at a total volume of 100μL per transwell. MCP-1 (30nM) or RANTES (12.8nM) were added to 100μL of HBSS containing 1% FBS and 1% penicillin/streptomycin and added to the top of the gel to initiate migration. Transwells were incubated for 48h at 37  C in 5% CO_2_ in a 24-well plate. In each well, 650μL of HBSS containing 1% FBS and 1% penicillin/streptomycin was added beneath the transwell insert to prevent drying of the gel during incubation. Experiment run in two independent replicates with wells of each respective cytokine plaved in triplicate. Migrating cells were detected using confocal microscopy (Leica TCS SP8), and results were analyzed with IMARIS 8.0 software. Wells showing migrated cells were used in statistical analysis, MCP-1 (n = 4) and RANTES (n = 3).

### qRT-PCR

Prior to RNA isolation, peritoneal macrophages were incubated overnight with LPS (10ng/mL) and PNU-282897 (30μM). Total RNA was extracted from thioglycolate-induced mouse peritoneal macrophages using the PureLink RNA Mini Kit (Invitrogen, Carlsbad, CA, USA). Reverse transcription was performed using the iScript cDNA Synthesis Kit (Bio Rad, Hercules, CA, USA). Roughly 0.8–1.0μg of cDNA was synthesized in a 20μL reaction volume, per the kit instructions. Real-time PCR reactions were set up in a 96-well qPCR plate using IQ SYBR Green Supermix (Biorad, Hercules, CA, USA) and run using the CFX96 Real Time Thermal Cycler fitted with a C1000 lid (BioRad). Each sample was plated in duplicate. Specific primers for each target were designed and are listed in Table 1 (Integrated DNA Technologies, Coralville, IA). Primer sequences were derived from previously published studies and verified using NCBI Blast and IDT Oligo Analyzer [27, 28]. Fold changes were normalized to GAPDH. Relative expression of each target was calculated using the Livak Method [29].

**Table 1.** Primers sequence used for qPCR.

Primer	Sequence (5’–3’)	References
αX forward	CTGGATAGCCTTTCTTCTGCT	[30]
αX reverse	GCACACTGTGTCCGAACTCA	[30]
αM forward	TCCGGTAGCATCAACAACAT	[31]
αM reverse	GGTGAAGTGAATCCGGAACT	[31]
αD forward	GGAACCGAATCAAGGTCAAGT	[32]
αD reverse	ATCCATTGAGAGAGCTGAGCTG	[32]
CCR2 forward	ACAGCTCAGGATTAACAGGGACTTG	[27]
CCR2 reverse	ACCACTTGCATGCACACATGAC	[27]
CCR5 forward	TCCGTTCCCCCTACAAGAGA	[28]
CCR5 reverse	TTGGCAGGGTGCTGACATAC	[28]
MCP-1 forward	TGGAGCATCCACGTGTTGGC	[33]
MCP-1 reverse	ACTACAGCTTCTTTGGGACA	[33]
RANTES forward	GCTTCCCTGTCATTGCTTGCTC	[34]
RANTES reverse	AGATGCCCATTTTCCCAGGACC	[34]
β1 forward	GTGACCCATTGCAAGGAGAAGGA	[35]
β1 reverse	GTCATGAATTATCATTAAAAGTTTCCA	[35]
GAPDH forward	AAGGTCATCCCAGAGCTGAA	[36]
GAPDH reverse	CTGCTTCACCACCTTCTTGA	[36]

### Trans-endothelial migration assay

Endothelial cells (HUVECS) were labeled using CellVue Claret (Sigma-Aldrich, St-Louis, MO) and incubated overnight on the membranes of 6.5mm transwell inserts with 8µm pores (Costar, Corning, NY) to form a monolayer. Non-adhered endothelial cells were gently washed out. WT and α7nAChR^−/−^ monocytes were isolated from bone marrow using magnetic-assisted cell sorting as described above in methods  "Isolation of Monocytes from Mouse Bone Marrow" section. Isolated monocytes were labeled using either PKH67 (green) or PKH26 (red), with colors switched to confirm that dye color does not influence the result. Stained monocytes were added on top of the endothelial cells. MCP-1 (30nM) or RANTES (12.8nM) were added to the bottom chamber to start migration, along with media containing 650μL of HBSS with 1% FBS and 2% penicillin/streptomycin to prevent drying. Each respective cytokine was plated in triplicate and the transmigration had three independent replicates. After 3 h, the migration was evaluated by confocal microscopy (Leica TCS SP8). Results were analyzed using IMARIS 8.0. Transwells with well-demarcated HUVEC monolayers were used for analysis, MCP-1 (n = 6), and RANTES (n = 9).

### Isolation of peripheral blood monocytes

To evaluate the potential changes in adhesion receptor expressions on circulation monocytes after the LPS challenge, monocytes were isolated from the peripheral blood of WT and α7nAChR^−/−^ mice. Male mice were injected with 8μg LPS per gram of body weight. After 3 h, mice were euthanized using Isothesia, and blood was collected in EDTA (200mM) coated syringes through cardiac puncture. Each mouse yielded 500–700μL of blood, which was diluted using an equal volume of balanced salt solution, prepared as instructed in the Cytiva Ficoll–Paque protocol. The monocytes were separated from whole blood using Ficoll–Paque 1.084 (Cytiva) according to manufacturer instructions. Isolated monocytes were then prepared for flow cytometry using anti-Ly6-C/FITC, anti-αM/PE-Cy7, anti-αL/APC, anti-L-selectin/PE, and anti-α4/PE across multiple samples.

## Statistical Analysis

Experimental data were analyzed using a two-tailed student’s *t* test. Results are given as mean ± SEM. Survival experiments were analyzed using the Kaplan Meier Method and Log Rank Test. Quantitative Real-Time PCR data were analyzed using the Livak Method. Values of p < 0.05 were considered to be statistically significant.

## Results

### 7nAChR deficiency results in increased mortality and decreased macrophage accumulation in the lungs, while activation of α7nAChR increases macrophage accumulation during endotoxemia

The anti-inflammatory effects of α7nAChR activation using small molecule agonists have been extensively studied in mice with endotoxemia and CLP [9, 18, 19]. For instance, administering a partially selective α7nAChR agonist, GTS-21, or choline—a product of acetylcholine degradation and a selective endogenous α7nAChR agonist suppresses circulating pro-inflammatory cytokine levels, which is linked to reduced NF-κB activation and increased survival in endotoxic mice and rates [11, 19]. However, the role of α7nAChR deficiency on survival in endotoxemia and macrophage migration into tissues was not previously investigated.

**Fig. 1:**
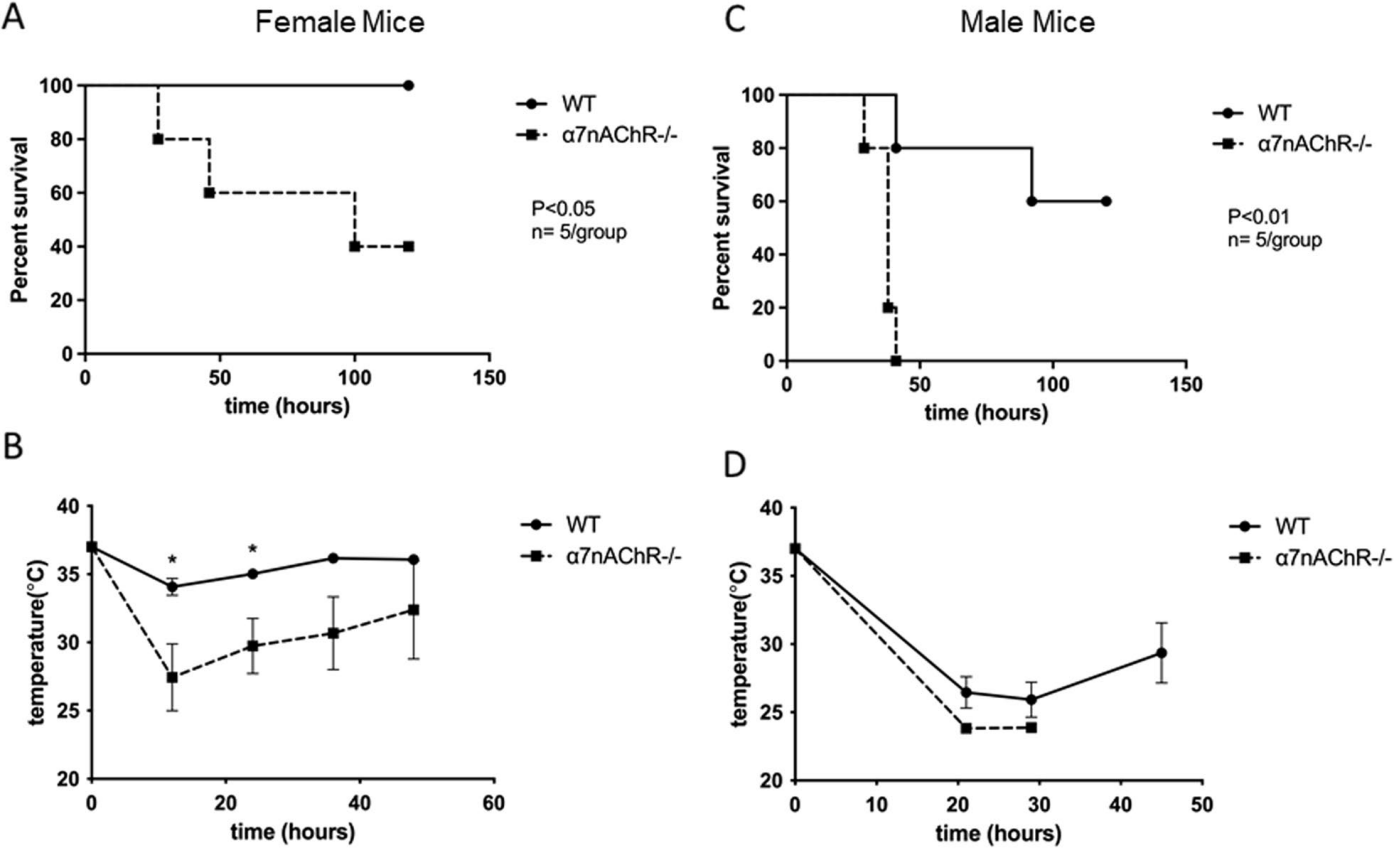
HYPERLINK "sps:id::fig1||locator::gr1||MediaObject::0"α7nAChR is protective during endotoxemia. **A** Survival curves after intraperitoneal administration of LPS to induce endotoxemia in female mice. (WT, *n* = 5; α7nAChR^−/−^, *n* = 5). **B** After injection of LPS, a decrease in body temperature to 21–27 ^o^C confirmed the development of endotoxemia. **C** Survival curves after LPS-induced endotoxemia in male mice. WT (*n* = 5) and α7nAChR^−/−^ (*n* = 5). **D** Body temperature drop in male mice. Statistical significance of survival curves was assessed by the Kaplan–Meier method. Temperature data are shown as mean±SEM, *P < 0.05

We first evaluated the impact of α7nAChR deficiency on morbidity during endotoxemia. Wild-type (WT) and α7nAChR^−/−^ mice, both male and female (n = 10/strain), were injected with LPS and morbidity was monitored for 4 days. α7nAChR deficiency significantly reduced the survival rates, exhibiting a similar pattern in both female (p < 0.05) and male (p < 0.01) mice (Fig. 1A, C). This reduction in survival correlated with a decrease in body temperature that indicated the severity of induced endotoxemia (Fig. 1B, D).

**Fig. 2:**
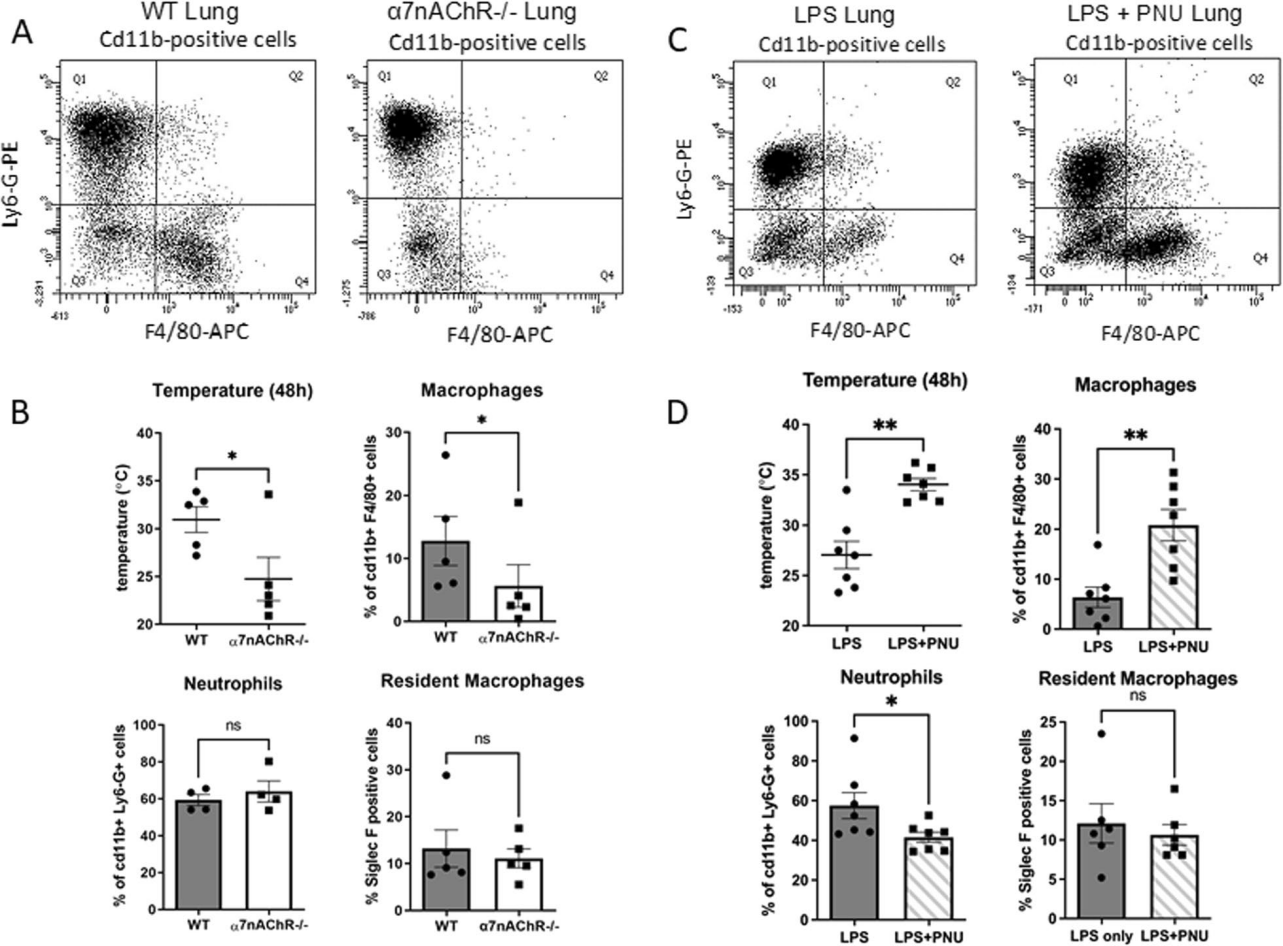
Macrophage accumulation in the lungs is affected by α7nAChR signaling. **A** WT and α7nAChR^−/−^ mice were injected with a sublethal dose of LPS. After 48h lungs were removed, digested, and analyzed using flow cytometry. CD11b-positive cells were selected and tested with antibodies against Ly6-G and F4/80 to identify neutrophils and macrophages, respectively. Results were analyzed and calculated using FACSDiva software and GraphPad Prism. **B** Plots representing the number of WT and α7nAChR^−/−^ macrophages (top right, *n* = 5/group), neutrophils (bottom left, *n* = 4/group), and resident macrophages (bottom right, n = 5/group) in digested lungs. Residents were identified as CD11b-F4/80 + Siglec F + . Body temperature at 48h is shown at top right (*n* = 7/group). **C** WT mice were injected with either a sublethal dose of LPS or 3mg/kg PNU-282987 followed by LPS 15 min later. The dose of LPS used was higher than for part A to generate more severe conditions for WT mice. After 48h lungs were removed, digested and analyzed using flow cytometry. CD11b positive cells and residents were selected and analyzed as above. Results were analyzed with FACSDiva software and calculated with GraphPad Prism. **D** Plots depicting the number of macrophages (top right, *n* = 7/group), neutrophils (bottom left, *n* = 7/group), and resident macrophages (bottom right, *n* = 6/group). Body temperature at 48h is shown at top left (*n* = 7/group). Statistical analysis was performed using a paired *t* test

The recruitment of monocytes/macrophages into the lungs can have a protective outcome during endotoxemia [26, 37–39]. Therefore, we reasoned that decreased macrophage accumulation in lungs could be responsible for the increased mortality of α7nAChR^−/−^ mice. To provide insight, age-matched WT and α7nAChR^−/−^ mice were injected with LPS (n = 5/group) and the severity of endotoxemia was verified by a drop in body temperature after 48 h (Fig. 2B). After 48 h, the lungs were collected and digested to analyze leukocyte populations using flow cytometry (Fig. 2A). Live cells were selected using a viability dye, and specific markers were used to evaluate different leukocyte subsets. Neutrophils were analyzed as CD11b + , Ly6-G + , and F4/80-. Monocyte-derived macrophages were identified as CD11b + , F4/80 + , and Ly6-G-, while alveolar macrophages were selected as F4/80 + , CD11b-, and Siglec F + . The gating strategies for monocyte-derived and alveolar macrophages are depicted in Additional file 1: Figs. S1 and S2, respectively. We observed that the percentage of monocyte-derived macrophages in the lungs of α7nAChR^−/−^ mice was significantly lower. The number of resident alveolar macrophages and neutrophils was not markedly different (Fig. 2B).

To assess the impact of α7nAChR activation on macrophage accumulation, we treated WT mice with either LPS and DMSO (vehicle) or LPS with α7nAChR agonist PNU-282987 (*n* = 7/group) (Fig. 2C). 3mg/kg PNU-282987 dissolved in DMSO or DMSO alone were administered 15 min prior to LPS injection as previously described [38]. Body temperature of mice receiving treatment with PNU-282987 was significantly higher at 48 h when compared to LPS only control. Using the same gating strategy for leukocyte subtypes, we observed that pre-treatment with PNU-282987 significantly increased the percentage of viable macrophages in the lungs and decreased the percentage of viable neutrophils (Fig. 2C, D). Interestingly, the proportion of viable resident alveolar macrophages in the lungs did not change with PNU-282987 treatment (Fig. 2D), but the absolute cell count was increased in PNU-282987-treated mice (Additional file 1: Fig. S2).

Multiple mechanisms can contribute to the reduced accumulation of macrophages in the lungs during endotoxemia, including decreased monocyte/macrophage infiltration, increased apoptosis and promoted necrosis. Analysis with Annexin V staining did not reveal a significant difference in the amount of apoptotic or necrotic (late apoptotic) macrophages (Additional file 1: Fig. S3A–C). Notably, the number of necrotic neutrophils was also similar (Additional file 1: Fig. S3D). Together, these findings suggest that α7nAChR-dependent macrophage accumulation is reliant on monocyte/macrophage migration.

### α7nAChR deficiency reduces the recruitment of monocyte-derived macrophages to the lungs during endotoxemia

**Fig. 3:**
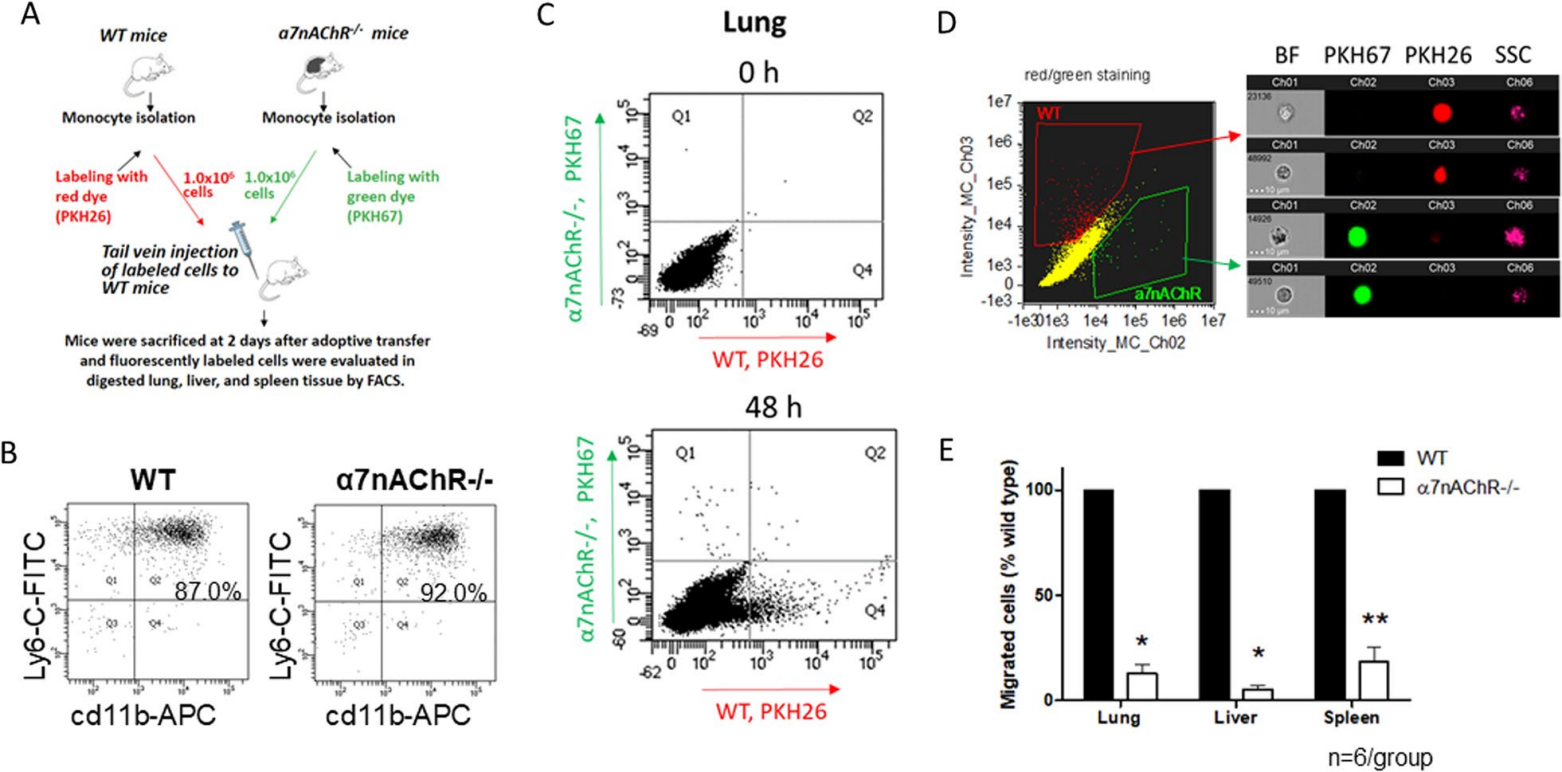
α7nAChR deficiency impedes the migration of macrophages to organs during LPS-induced endotoxemia.** A** Schematic representation of the experimental design. Monocytes were isolated from bone marrow of male WT and α7nAChR^−/−^ mice via MACS. Cells were labeled with red (WT) or green (α7nAChR^−/−^) fluorescent dyes, mixed in equal proportion and injected in tail vein of male WT recipient mice. After 48 h, the lung, liver and spleen were isolated, digested and analyzed using flow cytometry. **B** Representative dot plots of monocyte purity analysis. Isolated monocytes were labeled with anti-CD11b (APC) and anti-Ly6-G (FITC). Monocyte population is visible in Q2. **C** Representative results of flow cytometry analysis are shown. The leukocyte distribution in lungs before the adoptive transfer (Upper panel) and at 48 h after adoptive transfer and LPS administration (Lower panel) are presented. Data were analyzed using FACSDiva software. Migrated WT macrophages (red) were detected in Quadrant 4; α7nAChR^−/−^ macrophages (green) were detected in Quadrant 1. **D** Imaging flow cytometry of labeled macrophages. (BF = bright field, SSC = side scattering). **E** Bar graphs representing the amount of WT and α7nAChR-deficient macrophages detected in lungs, liver, and spleen by flow cytometry, (*n* = 6). Statistical analysis was performed using student’s *t* test. *P < 0.05, **P < 0.01

To further assess the role of α7nAChR in monocyte/macrophage migration, we conducted an in vivo adoptive transfer tracking experiment in the same model of endotoxemia to examine monocyte recruitment to the lungs, liver, and spleen. Fluorescently labeled WT and α7nAChR^−/−^ monocytes were injected intravenously to recipient mice, as depicted in Fig. 3A. Monocytes were isolated from bone marrow by negative selection (87–92% purity, Fig. 3B) and labeled with either green PKH67 (α7nAChR^−/−^) or red PKH26 (WT) fluorescent dyes. WT and α7nAChR^−/−^ monocytes were mixed in equal proportions and injected into recipient mice immediately followed by a sub-lethal dose of LPS. The cell mixture proportion was validated by fluorescent microscopy of a Cytospin slide (Additional file 1: Fig. S4A). After 48 h, the lungs, spleen and liver were digested for flow cytometry analysis to identify labeled, migrated macrophages (Fig. 3C). Flow cytometry data for the liver and spleen are shown in Additional file 1: Fig. S5. In addition, Imaging Flow Cytometry (Amnis) was employed to verify macrophage integrity, morphology and labeling (Fig. 3D). Our analysis revealed that lungs and other organs of WT recipient mice accumulate a significantly lower number of donor α7nAChR^−/−^ macrophages compared to donor WT macrophages (Fig. 3E).

**Fig. 4:**
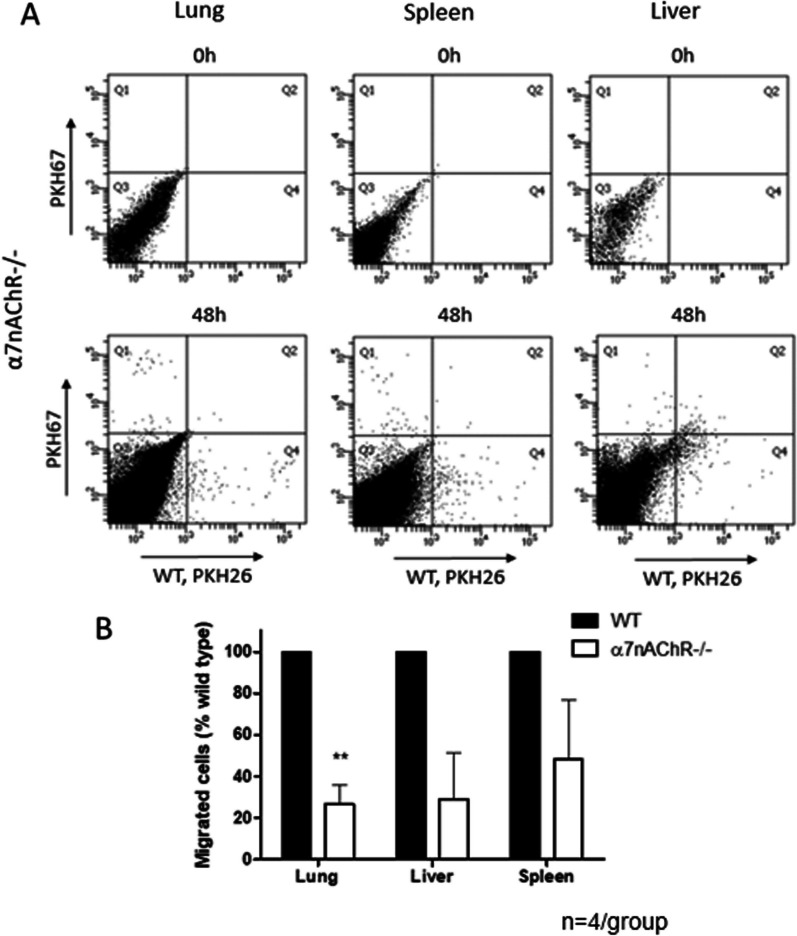
Effect of α7nAChR deficiency on migration does not depend on other cell types. **A** Representative dot plots of flow cytometry showing migrated red (WT) and green (α7nAChR^−/−^) monocytes in male α7nAChR^−/−^ recipient mice. The leukocyte distribution in lungs before the adoptive transfer (Upper panel) and at 48 h after adoptive transfer and LPS administration (lower panel) are presented. **B** Bar graphs representing the amount of WT and α7nAChR-deficient macrophages detected in organs by flow cytometry. The experimental setup is same, as depicted in Fig. 3A, using 8-week-old male and female α7nAChR^−/−^ recipients instead of WT (*n* = 4). Statistical analysis was performed using a student’s *t* test. **P < 0.01

An overall similar trend was observed in the adoptive transfer of WT and α7nAChR^−/−^ monocytes to α7nAChR^−/−^ recipient mice (Fig. 4) that indicates that other cell types expressing α7nAChR do not significantly influence the migration of monocytes/macrophages to tissue. Therefore, the expression of α7nAChR on the monocyte/macrophage surface is critical for effective cell migration.

It is worth noting that PKH67 and PKH26 are widely used membrane stains and that we have previously demonstrated that switching red and green dye colors does not impact the results of our adoptive transfer experiments [32, 40, 41]. In an additional experiment, the adoptive transfer approach was performed using only WT monocytes labeled with PKH26 or PKH67 dyes, and no significant difference was observed in the number of PKH26- or PKH-67-labeled WT macrophages in the inflamed tissue (Additional file 1: Fig. S4C,D).

### Trans-endothelial migration of monocytes is not affected by α7nAChR deficiency

Trans-endothelial migration is a critical step in the recruitment of monocytes from the blood into inflamed tissues. We tested whether α7nAChR deficiency affects this process. A diagram depicting the experimental setup is shown in Additional file 1: Fig. S6A. After a 3-h incubation, cells that had crossed the endothelial cell monolayer were visualized using confocal microscopy, and images were reconstructed using IMARIS 8.0 software (Additional file 1: Fig. S6B). Notably, we observed no significant difference in the number of translocated WT and α7nAChR^−/−^ monocytes in response to MCP-1 or RANTES (Additional file 1: Fig. S6C).

Because αM, αL, α4, and L-selectin are adhesion receptors and crucial in the trans-endothelial migration process, we examined their expression on the cell surface of mouse peripheral blood monocytes using flow cytometry. Monocytes were gated based on Ly6-C positivity before assessing the receptors relative expression in mean fluorescence intensity (MFI). We found no significant difference in MFI or the percentage of αL + , L-selectin + , α4 + , or αM + cells between WT and α7nAChR^−/−^ mice (Additional file 1: Fig. S6D). These findings confirm that α7nAChR deficiency does not impact the ability of isolated monocytes to migrate across an endothelial monolayer.

### α7nAChR deficiency reduces the migratory ability of macrophages in a 3D fibrin matrix along a chemokine gradient

**Fig. 5.**
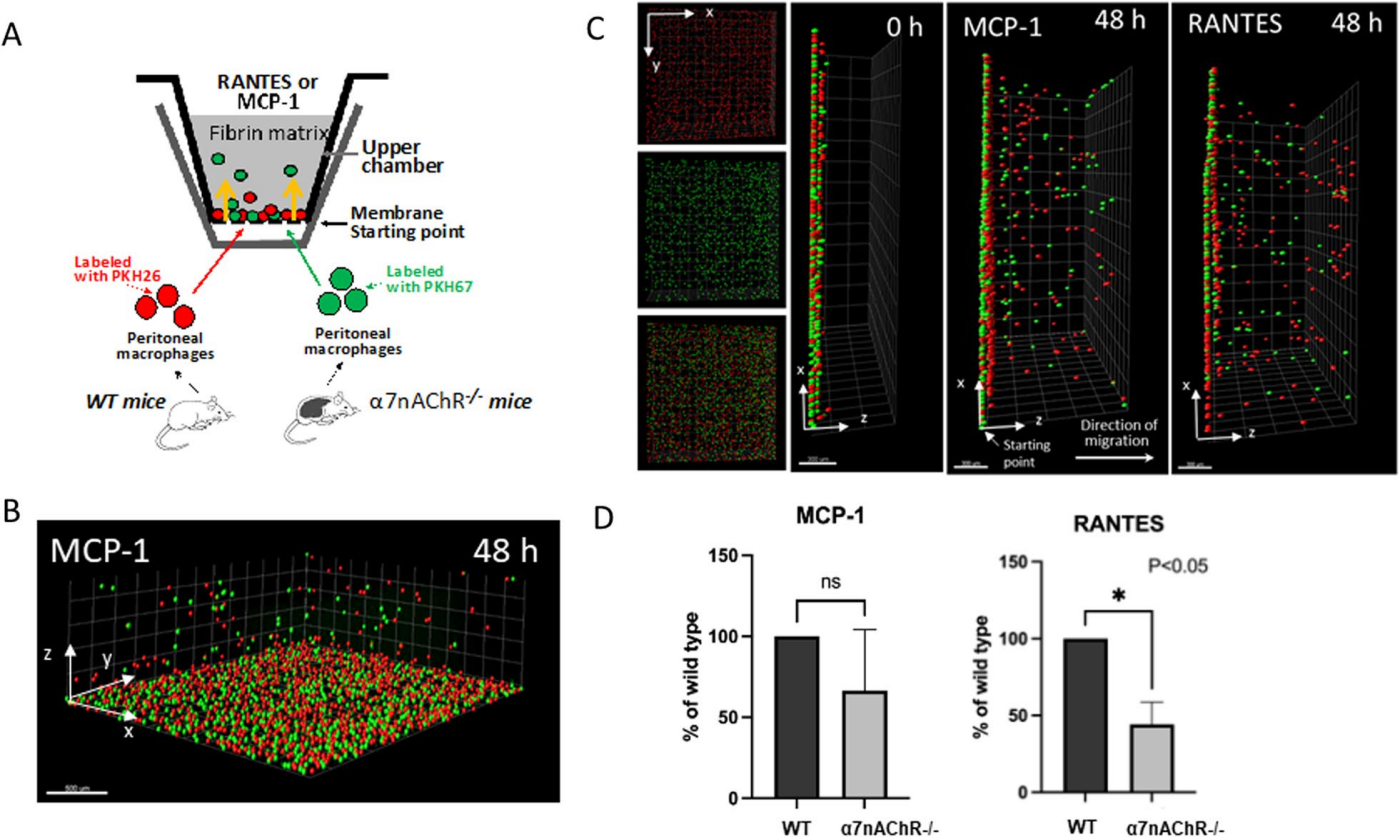
3-D migration of peritoneal macrophages along MCP-1 and RANTES gradients. **A** Schematic drawing of experimental setup within a Corning transwell insert, with yellow arrows indicating the direction of macrophage migration. WT macrophages were labeled red (PKH26) and α7nAChR^−/−^ macrophages were labeled green (PKH67) before being added to the membrane. Migration was initiated using RANTES (12.8nM) or MCP-1 (30nM) in medium added to the top of the fibrin gel. **B** 3-D view of labeled macrophages migrating inside the fibrin gel after 48 h. **C** IMARIS 8.0 reconstruction of WT (red) and α7nAChR^−/−^(green) macrophages before the initiation of migration and after 48-h incubation. Left shows the top view of individual and combined channels. Center, side view showing starting point at 0h. Right, side view showing macrophages migrating along MCP-1 or RANTES gradients. **D** The number of macrophages migrating greater than 80μm was analyzed as a percentage of WT (MCP-1, *n* = 4; RANTES, *n* = 3). Statistical analysis was carried out using a student's *t* test. *P < 0.05

To support our in vivo findings, we conducted an in vitro experiment to investigate the impact of α7nAChR on macrophage migration within a 3D matrix. WT and α7nAChR^−/−^ macrophages were allowed to migrate through a 3D fibrin matrix within a transwell insert in response to a gradient of either MCP-1 or RANTES. A schematic diagram of the experimental setup is illustrated in Fig. 5A. Equal proportions (7.5 × 10^5^) of fluorescently labeled WT (PKH26, red) and α7nAChR^−/−^ (PKH67, green) macrophages were placed on the membrane of the insert before the addition of the matrix. MCP-1 (30nM) or RANTES (12.8nM) were then added to the top of the fibrin matrix to initiate migration. After 48 h of incubation at 37 °C and 5% CO_2_, migrated cells were visualized using confocal microscopy (Fig. 5B, C) and images were reconstructed using IMARIS 8.0 software.

α7nAChR^−/−^ macrophages exhibited significantly reduced migration in response to a RANTES gradient compared to WT macrophages (Fig. 5D). Although the response to an MCP-1 gradient showed a similar pattern, it did not reach statistical significance. These findings complement our in vivo results and support the suggestion that α7nAChR deficiency may alter the expression of chemokine receptors and/or adhesive receptors.

### α7nAChR deficiency markedly reduces relative mRNA levels of integrins αM and αX

Based on the results obtained from our 3D migration assay and tracked adoptive transfer experiment, we hypothesized that the expression of chemokine receptors and adhesive receptors may be altered in α7nAChR-deficient macrophages, leading to a decrease in their migration. We investigated the expression levels of adhesion receptors from β2 integrin family: αM, αD, αX and directly from β1 integrin (which forms the complex with several α subunits, including α2, α3, α4, α5) as these receptors play a role in macrophage migration by interacting with extracellular matrix proteins. Chemokine receptors CCR2 and CCR5 were also examined, as they are major receptors in macrophage chemotaxis, as well as the respective receptors for MCP-1 and RANTES, the chemokines used in our 3-D migration assay. To assess the mRNA levels of these selected receptors, we performed quantitative real-time polymerase chain reaction (qRT-PCR) using thioglycolate-induced peritoneal macrophages that were incubated overnight with LPS (10ng/mL) and PNU-282987 (30μm). Total RNA was extracted from the macrophage lysate and used for qRT-PCR analysis. The specific primers used for detecting αM, αD, αX, CCR2, and CCR5 are listed in Table 1 of the methods section.

**Fig. 6:**
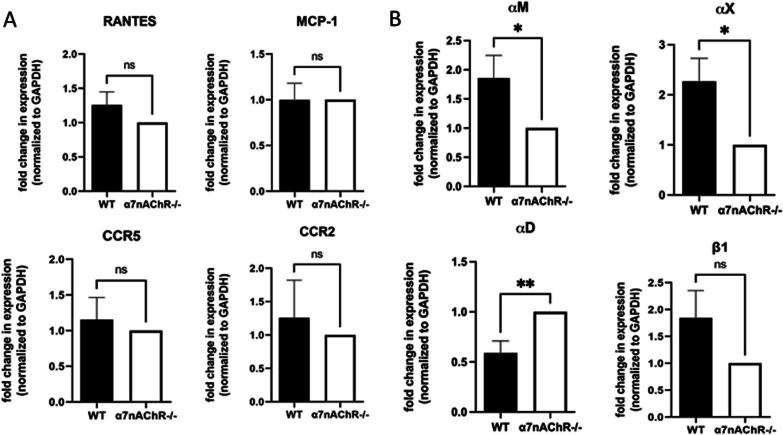
Quantitative real-time PCR of chemokines and surface receptors. **A** Peritoneal macrophages were isolated from WT and α7nAChR^−/−^ mice and subsequently incubated with LPS (10ng/mL) and PNU (30μM) overnight before isolation of RNA and production of cDNA. Plots show relative mRNA levels of CCR2 and its ligand MCP-1, and CCR5 and its ligand RANTES. **B** Cells were prepared for qRT-PCR identically to part A. Plots showing relative mRNA levels of integrin subunits αM, αX, αD, and β1. Experiment had eight independent replicates. Data were analyzed using the Livak Method. Statistical analysis was performed with a student's *t* test. *P < 0.05, **P < 0.01

α7nAChR-deficient macrophages exhibited similar relative mRNA levels of CCR2 and CCR5 compared to WT controls (Fig. 6A). However, the relative mRNA levels of integrins αM and αX in α7nAChR-deficient macrophages showed a statistically significant decrease compared to WT controls (Fig. 6B). Integrin subunit ß1 did not show any significant changes in α7nAChR deficient mice (Fig. 6B). Furthermore, we examined the relative mRNA levels of the corresponding chemokines, MCP-1 and RANTES, which are secreted by macrophages to attract additional leukocytes to the site of inflammation. The transcription of RANTES and MCP-1 remained relatively unchanged by α7nAChR deficiency (Fig. 6A).

These findings indicate that the deficiency of α7nAChR leads to significant decreases in the relative mRNA levels of αM and αX in macrophages. These alterations in receptor expression may contribute to the impaired migration observed in α7nAChR-deficient macrophages. In addition, the similar relative mRNA levels of CCR2, CCR5, RANTES, and MCP-1 rule out the possibility of an altered response in a7nAChR deficient macrophages via these chemotactic mechanisms.

### α7nAChR deficiency reduces expression of αM at the cell surface of adoptively transferred monocytes

**Fig. 7:**
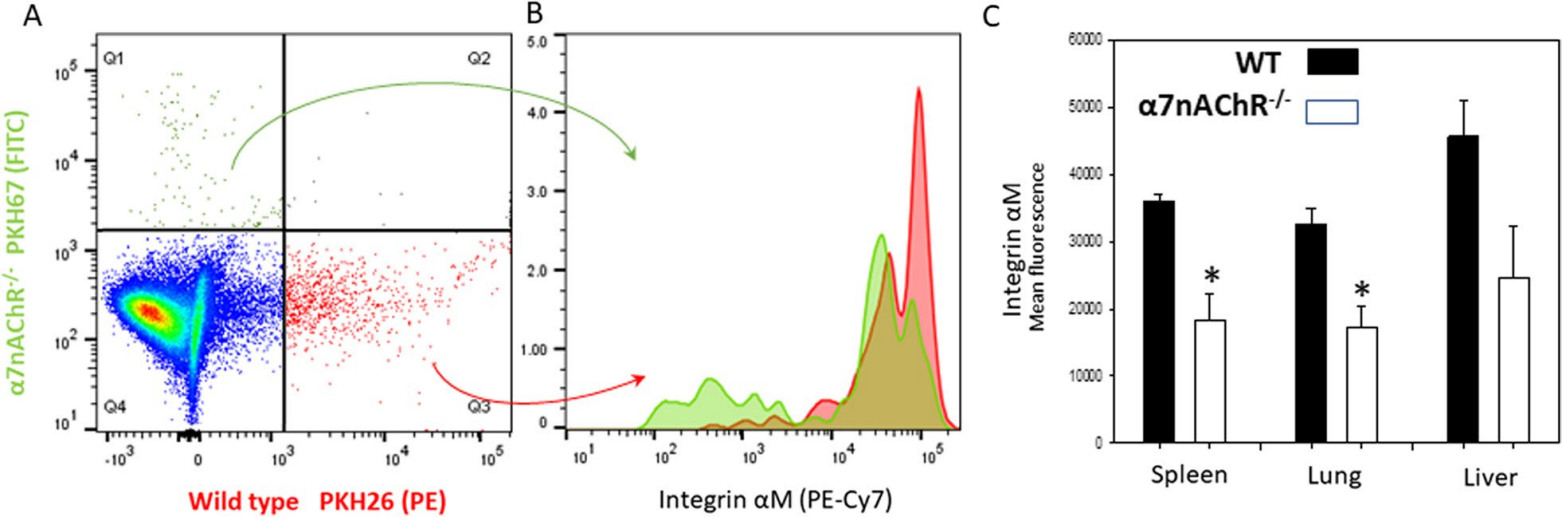
Surface expression of integrin αM on migrating WT (red) and α7nAChR^−/−^ (green) macrophages.** A** Representative flow cytometry dot plot showing migrated WT (red) and α7nAChR^−/−^ (green) macrophages, in quadrant 3 and quadrant 1, respectively. **B** Histogram overlay of αM fluorescence, colors correspond to cell staining in part A. Data were analyzed using FlowJo software. **C** Bar graphs representing the amount of WT and α7nAChR-deficient macrophages detected in organs by flow cytometry. (*n* = 4). Statistical analysis was performed using student’s *t* test. *P < 0.05

The macrophage expression of integrin αX is dramatically lower when compared with the level of integrin αM [42] (Additional file 1: Fig. S7). Due to its lower expression, αX is unlikely to significantly contribute to macrophage migration. Therefore, we focused primarily on αM. To verify our qPCR results, we assessed the expression levels of αM on adoptively transferred fluorescently labeled WT (PKH26) and α7nAChR^−/−^ (PKH67) macrophages in lungs at 48 h after intraperitoneal injection of LPS (Fig. 7A–C). The expression of αM on adoptively transferred α7nAChR-deficient (green) monocytes was significantly reduced compared to WT (red) monocytes (Fig. 7C). This finding suggests that α7nAChR deficiency may affect the mesenchymal mode of macrophage migration, as αM plays a crucial role in the movement and adhesion of macrophages in the extracellular matrix.

### Adoptive transfer of WT macrophages to α7nAChR-deficient recipients leads to the partial rescue of phenotype

Since α7nAChR expression on macrophages has a protective effect during endotoxemia, we reasoned that injecting WT monocytes into α7nAChR-deficient mice could potentially rescue the protective phenotype of α7nAChR and improve survival. Conversely, we also investigated whether injecting α7nAChR-deficient monocytes to WT recipients would adversely affect survival.

**Fig. 8:**
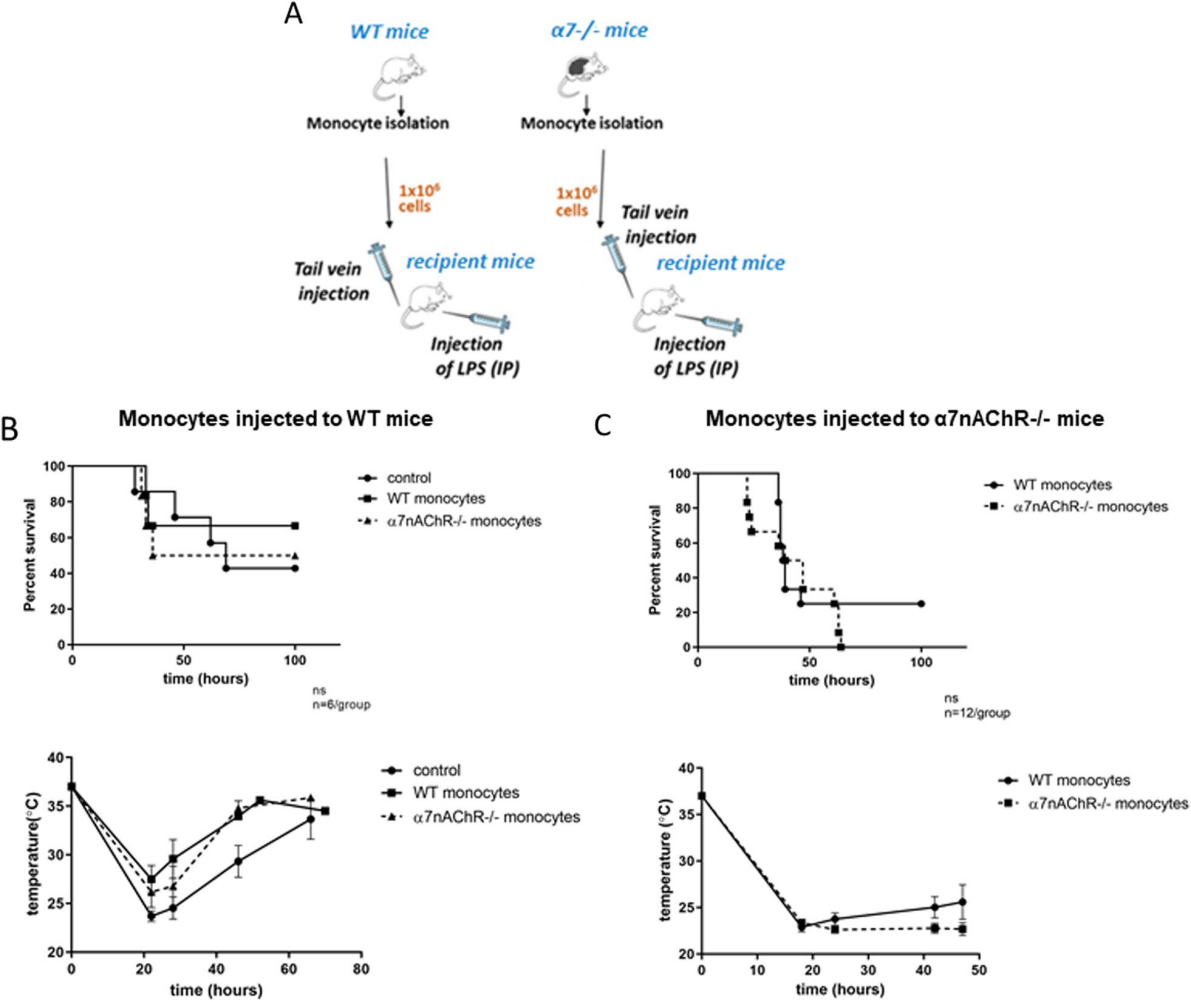
Survival of WT and α7nAChR^−/−^ mice injected with monocytes during LPS-induced endotoxemia. **A** Graphical representation of experimental setup. Recipient mice are either WT (part B) or α7nAChR^−/−^ (part C). Control mice were given LPS only, with no monocytes. **B** Survival curve and temperature graph of WT recipients receiving WT, α7nAChR^−/−^, or no monocytes (control) intravenously before a sub-lethal intraperitoneal dose of LPS. 8–10-week-old WT male and female mice were used as recipients (*n* = 6/treatment group). **C** Survival curve and temperature graph of α7nAChR^−/−^ recipients receiving either WT or α7nAChR^−/−^ monocytes intravenously before a sub-lethal intraperitoneal dose of LPS. 8–10-week-old α7nAChR^−/−^ male and female mice were used as recipients (*n* = 9/treatment group). For survival curves, statistical significance was assessed by the Kaplan–Meier method. Temperature graphs report mean temperature and standard error.

First, we evaluated the potential effect of α7nAChR-deficient monocytes injected to WT recipient mice. WT mice were divided into two groups (n = 6/group) and injected with WT or α7nAChR^−/−^ monocytes intravenously 5 min prior the induction of endotoxemia. In addition, a third group was injected with the same concentration of LPS without adoptively transferred monocytes. A schematic diagram illustrating the experimental setup is shown in Fig. 8A. Body temperature and morbidity of the mice were monitored twice daily for 4 days. We did not find significant differences in survival between the three groups (Fig. 8B).

To test a potential protective mechanism of WT monocytes, we divided α7nAChR^−/−^ recipient mice into two groups (n = 12/group) and administered unlabeled WT or α7nAChR^−/−^ monocytes intravenously prior to inducing endotoxemia. The mice were monitored for 4 days for changes in body temperature and morbidity (Fig. 8C). All α7nAChR-deficient recipient mice injected with α7nAChR^−/−^ monocytes died within 60 h, while the same strain injected with the WT monocytes demonstrated a modest improvement in survival (25%) (Fig. 8C). These results demonstrate a partially protective effect of WT monocyte transfer to α7nAChR-deficient recipients.

## Discussion

Here we showed that genetic α7nAChR deficiency is associated with reduced macrophage migration to the lungs during murine endotoxemia and specific pharmacological activation of this receptor results in increased macrophage migration. These observations indicate a previously unrecognized role for the α7nAChR, a key peripheral component of the cholinergic anti-inflammatory pathway, in mediating macrophage migration during acute inflammation. In parallel, α7nAChR deficiency results in increased mortality of mice during endotoxemia, which indicates a tonic protective function of the α7nAChR in inflammation. These findings identify macrophage migration as an important mechanism contributing to the physiological cholinergic regulation of inflammation. Additional mechanistic insight substantiates this notion, revealing that the expression of integrin αMβ2 is reduced on α7nAChR-deficient monocyte-derived macrophages, indicating its potential role in α7nAChR-mediated macrophage migration.

Macrophages are essential players in innate immunity that may have a protective or pathological contribution to the development of inflammatory diseases [23]. Macrophage phenotype, tissue distribution, molecular environment, and disease stage define the outcomes of macrophage function. Inhibition of pro-inflammatory cytokine secretion by macrophages was the major anti-inflammatory function reported for α7nAChR [10, 43, 44]. Pioneering work from Kevin Tracey’s group revealed that α7nAChR activation blocks the nuclear translocation of NF-κB, a master transcription factor for multiple pro-inflammatory genes that generate inflammatory responses [6, 12, 45–47]. However, other potential mechanisms may have a significant contribution to the α7nAChR-mediated macrophage response.

Despite a well-characterized protective role of α7nAChR in endotoxemia and CLP sepsis, the direct effect of α7nAChR-deficiency on survival during endotoxemia was not investigated previously. Our results of α7nAChR^−/−^ mice compared with WT clearly demonstrate the detrimental impact of α7nAChR deficiency on survival during murine endotoxemia and complement previous observations that administration of the α7nAChR agonists GTS-21 or choline improve the survival of mice during endotoxemia and CLP sepsis [18, 19]. The significant drop of body temperature at 24 h after LPS-injection is an important pathophysiological effect of endotoxemia. Consequently, body temperature analysis was implemented as a reliable verification of endotoxemia severity and progression in our in vivo experiments.

Previous studies reported that the accumulation of macrophages in the lungs during sepsis can have a protective function [21, 22]. In contrast, the accumulation of neutrophils is a characteristic feature of sepsis-induced acute lung injury and is associated with poor outcomes [37, 48]. One of the mechanisms by which macrophages provide protection is through the control of inflammation via efferocytosis of activated neutrophils (Bailey et al. 2021). Here, we evaluated the effect of α7nAChR activation using a specific agonist on macrophage accumulation in lungs. Previous studies demonstrated the potent anti-inflammatory effects of α7nAChR activation by agonists, such as GTS-21 and PNU-282987 in murine models of systemic inflammation and sepsis [18, 19, 38, 44, 49]. We observed that wild-type mice treated with the agonist PNU-282987 exhibited a significant increase in the number of monocyte-derived macrophages and body temperature, along with a decrease in neutrophil numbers when compared to untreated mice. This finding is consistent with data reported by Huston et al. where nicotine treatment decreased the number of neutrophils accumulated in carrageenan-filled air pouches, as compared to controls [50].

Consistent with our α7nAChR activation approach, we observed a significant reduction in the number of macrophages in the lungs of α7nAChR-deficient mice during endotoxemia. These data were supported by the decrease in body temperature in α7nAChR-deficient mice which indicates the greater severity of systemic inflammation in these animals.

To provide additional insights in our study, we evaluated in vivo migration by monitoring fluorescently labeled, adoptively transferred monocytes/macrophages in the model of endotoxemia [32, 40, 41, 51]. We employed an internal control within each recipient mouse by injecting an equal number of monocytes from both WT and α7nAChR^−/−^ donors, facilitating direct comparison between the two monocyte types. An additional adoptive transfer tracking experiment was performed using α7nAChR-deficient recipients. Both experiments revealed the same pattern: more WT monocytes were detected in the lungs, liver, and spleen when compared to α7nAChR-deficient monocytes. In addition, to confirming the outcome of the experiment with WT recipients, the repetition with α7nAChR-deficient recipients suggests that the enhanced migration of WT monocytes does not depend on the expression of α7nAChR on other cell types. In both setups, the quality of the isolated donor monocytes was validated using flow cytometry, where a purity of 87–92% was confirmed.

To address any potential influence of fluorescent dyes on macrophage migration in vivo, we conducted a separate experiment comparing the migration of equal numbers of WT monocytes labeled with either PKH26 (red) or PKH67 (green) and found that both red and green-labeled WT macrophages exhibited similar motility when migrating towards inflamed tissue. These results provided evidence that the fluorescent dyes themselves do not significantly affect macrophage migration.

To demonstrate the direct involvement of α7nAChR in macrophage migration, we conducted in vitro 3D migration assays, a well-developed technique that we have previously used [32, 40, 41]. Namely, our experimental setup provided a comprehensive assessment of macrophage migration, wherein monocyte-derived WT and α7nAChR^−/−^ macrophages, labeled with different fluorescent dyes, migrated through a fibrin matrix against gravity in the presence of a chemokine gradient. By including two types of fluorescently labeled cells (WT and α7nAChR^−/−^) within the same matrix, we reduced data variability and enabled accurate calculation of the migration ratio between control and knockout macrophages in each sample. Within the fibrin matrix, we observed that α7nAChR-deficient macrophages exhibited reduced effectiveness in migrating along both RANTES and MCP-1 gradients compared to WT macrophages.

In comparison with our experimental protocol, previous studies that attempted to evaluate the contribution of α7nAChR to macrophage migration utilized macrophage-like cell lines, wild-type cell phenotype (no α7nAChR-knockout), and the most importantly, a simplified 2D transmigration setup without chemokine gradients or protein coatings. For example, the ability of the α7nAChR agonists (PHA-543613 and varenicline) to decrease migration of RAW264.7 cells was demonstrated by testing cell transmigration through uncoated trans-well membranes (Boyden chambers) without a chemokine gradient [52, 53]. Similar results were obtained by others who showed that acetylcholine can inhibit LPS-induced RAW264.7 cell migration. This study suggested that the inhibition of migration was attributed to the blocking of MMP-9 expression [54]. MMPs play a role in 3D macrophage migration through the extracellular matrix (ECM) by degrading ECM proteins and creating space for cell movement. However, it should be noted that the presented experiments do not directly verify this hypothesis, as the proposed model using un-coated transwells does not involve MMP-mediated ECM degradation. In this model, macrophages transmigrate via an 8-µm pore-size membrane without immobilized ligands and chemokine gradients, where cell motility is mostly regulated by gravity and diffusion. Therefore, the evaluation of the role of α7nAChR in macrophage migration remained incomplete. In this study, we provided advanced characterization by implementing an improved experimental design and methodology.

In addition to migration through the extracellular matrix, trans-endothelial migration is another crucial step in the recruitment of leukocytes during inflammation. Our findings revealed no significant difference in the transmigration of WT and α7nAChR^−/−^ monocytes across an endothelial monolayer in response to either MCP-1 or RANTES. These data were supported by the similar expression of integrins αL, αM, α4, and L-selectin on WT and α7nAChR^−/−^ mouse peripheral blood monocytes. These molecules are key adhesion receptors in the process of adhering to, and migrating across, the endothelial wall. Based on these results, we concluded that trans-endothelial migration does not contribute to the differential migration observed between WT and α7nAChR^−/−^ monocytes/macrophages.

Reduced expression of integrin αX and integrin αM at the transcriptional level in α7nAChR^−/−^ macrophages provides a potential mechanistic explanation for their reduced migration. Integrin αMβ2 is a crucial adhesive receptor for the recruitment of monocytes and migration of macrophages through the extracellular matrix. Integrin αXβ2 possesses multiple regulatory functions on macrophages [55, 56], but has a limited effect on macrophage migration due to relatively low level of expression on macrophage subsets. Therefore, the decreased αM mRNA and protein levels in α7nAChR^−/−^ macrophages suggest that their impaired migration may be due to altered adhesion.

In contrast to these findings, we observed an increased expression of integrin αDβ2 in α7nAChR^−/−^ macrophages. Integrin αDβ2 is significantly upregulated on pro-inflammatory (M1-like) macrophages in vivo and in vitro and contributes to the development of various chronic inflammatory diseases, such as atherosclerosis and diabetes [32, 41]. Importantly, previous studies have shown that activation of α7nAChR leads to macrophage polarization toward the M2 phenotype [38, 39, 57]; therefore, α7nAChR deficiency should be associated with M1 phenotype, where integrin αDβ2 is upregulated. Based on the levels of αMβ2 and αDβ2 expression on different macrophage subsets, it was suggested that αMβ2 is involved in macrophage migration to and from the sites of inflammation, while αDβ2 plays a role in the retention of pro-inflammatory, M1-polarized macrophages at the sites of chronic inflammation [40]. Therefore, the modest upregulation of the low-expressed αDβ2 on monocyte-derived macrophages may have a limited impact on macrophage migration but highlights the potential pathological role of integrin αDβ2 in α7nAChR-deficient mice during the development of atherosclerosis and diabetes [58–60].

Interestingly, the injection of WT monocytes did not completely rescue the phenotype of α7nAChR-deficient mice, resulting in only partial improvement in survival. It could be that this incomplete rescue is attributed to the already overwhelming NF-κB activation present in α7nAChR^−/−^ mice; thus, adding a population of WT monocytes may not be sufficient to reverse the deleterious effects caused by α7nAChR deficiency.

The recent discovery of CHRFAM7A, a human-specific dominant negative regulator of α7nAChR function expanded our understanding of how the cholinergic anti-inflammatory pathway is regulated in humans [61, 62]. The translated protein CHRFAM7A (dupα7) lacks the acetylcholine binding site, leading to reduced α7 receptor activity. In a study using peripheral blood mononuclear cells from septic patients, Cedillo et al. reported that CHRFAM7A has an inverse relationship with disease severity as well as cholinergic anti-inflammatory pathway activity, where patients with higher expression of CHRFAM7A had poorer prognoses [63]. THP-1 human monocytic cells transfected with dupα7 demonstrated a reduced migration, colony formation, and chemotaxis toward MCP-1 [64]. Therefore, the reduced α7 receptor activity inhibits macrophage migration, aligning with our observation that α7nAChR deficiency has a negative impact on the migration of mouse macrophages. Despite the translational incongruency, basic research of α7nAChR and the cholinergic anti-inflammatory pathway thus far in rodents, primary human monocytes, and cell lines has provides invaluable insights and presented therapeutic opportunities for the treatment of sepsis.

Based on our results, we propose that α7nAChR deficiency leads to reduced migration of macrophages to the lungs and other inflamed organs, thereby impairing the clearance of recruited neutrophils through efferocytosis. Consequently, neutrophils present in the tissues of α7nAChR^−/−^ mice secrete pro-inflammatory cytokines. Furthermore, the failed recruitment of α7nAChR-deficint monocytes results in their accumulation in the bloodstream and the secretion of pro-inflammatory cytokines via NF-κB-related mechanisms [65, 66]. Collectively, these processes contribute to an increased cytokine storm and higher mortality rate.

## Conclusions

Our findings indicate that the cholinergic anti-inflammatory pathway, specifically the α7nAChR, plays a crucial role in regulating the migration and accumulation of macrophages at inflammation sites. We present evidence that α7nAChR is not only protective during endotoxemia but also essential for efficient monocyte/macrophage trafficking. Using α7nAChR-deficient mice or stimulating WT mice with PNU-282987, we demonstrate that the α7nAChR supports the recruitment of monocyte-derived macrophages to the lungs in vivo and their migration in a 3D matrix in vitro. α7nAChR deficiency adversely affects migration, which is associated with reduced the levels of integrin αMβ2, a critical integrin involved in various stages of the leukocyte migration process. In summary, our findings provide novel insights into α7nAChR-mediated monocyte/macrophage migration to inflamed tissues, expanding the clinical possibilities for septic patients.

### Supplementary Information

**Additional file 1.** Supplementary figures.

## Data Availability

The data sets used and/or analyzed during the current study are available from the corresponding author on reasonable request.

## Abbreviations

α7nAChR: Alpha 7 nicotinic acetylcholine receptor
VNS: Vagal nerve stimulation
CLP: Cecal ligation and puncture
WT: Wild type
LPS: Lipopolysaccharide
ECM: Extracellular matrix
