# Supplementary material for: Cholinergic signaling via the α7 nicotinic acetylcholine receptor regulates the migration of monocyte-derived macrophages during acute inflammation
Source: J Neuroinflammation. 2024 Jan 4;21:3. doi: 10.1186/s12974-023-03001-7 (PMC10765732; doi:10.1186/s12974-023-03001-7)
Supplement: Supplementary file 1 — Additional file 1. Supplementary figures. [file 12974_2023_3001_MOESM1_ESM.pdf]

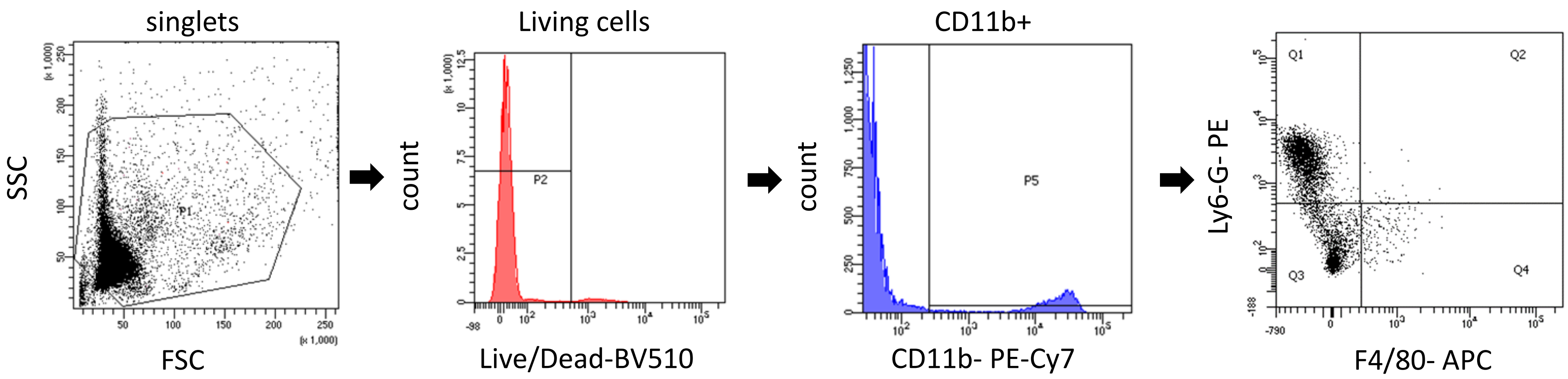

Supplemental Fig. 1. Gating strategy for Fig. 2A,C. To analyze alive macrophages and neutrophils, cells were gated by size to select singlets from all events (P1). Next, living cells were selected by gating cells negative for yellow fixable dye, BV510 (P2). From living cells, all CD11b+ events were selected (P5). Lastly, CD11b+ alive cells were analyzed using a dot plot, Ly6-G (neutrophil marker) VS F4/80 (macrophage marker). A quadrant gate was placed in reference to the negative control, so that neutrophils could be quantified in Q1 (Ly6-G-PE) and macrophages in Q4 (F4/80-APC).

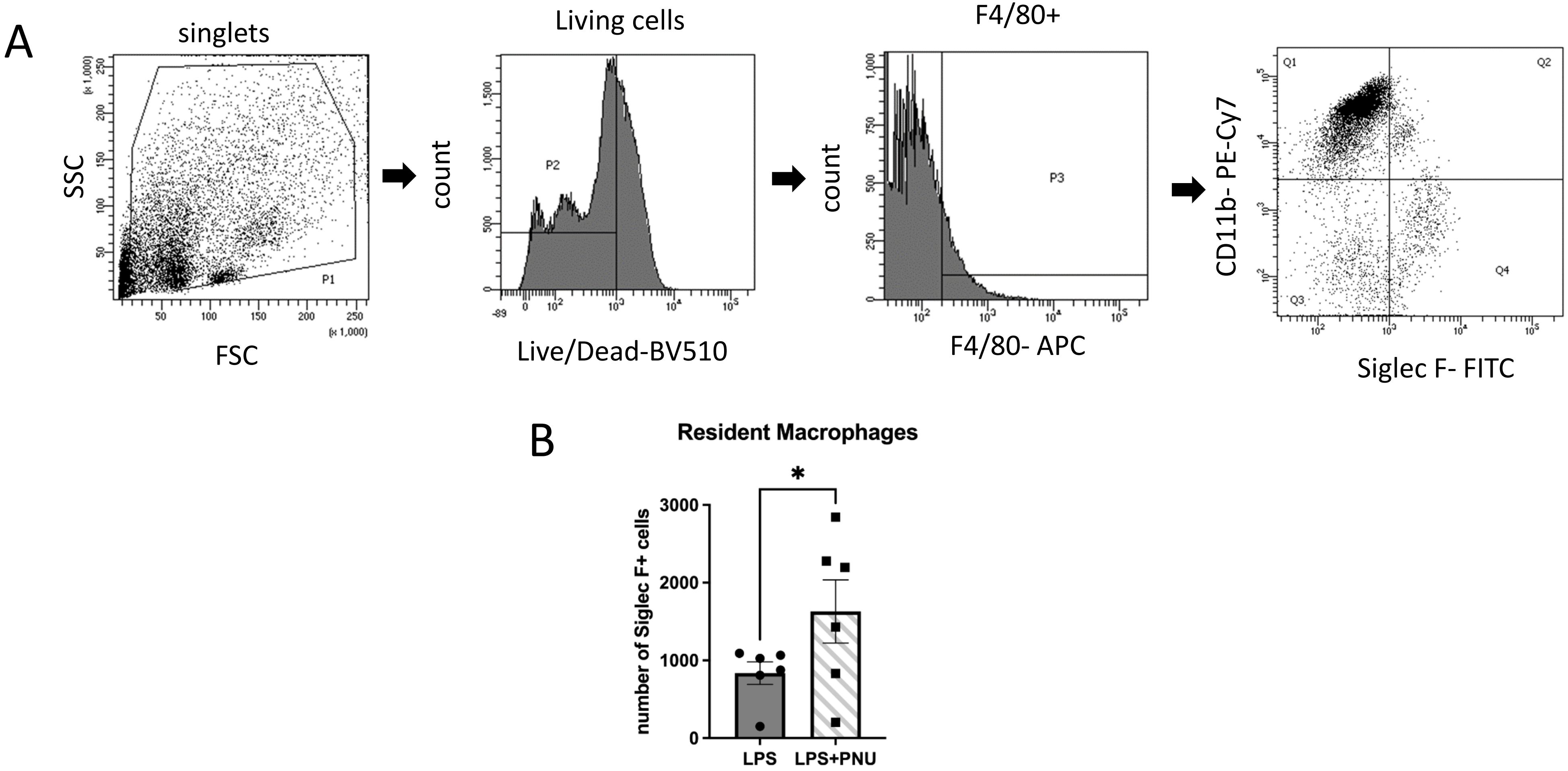

Supplemental Fig. 2. PNU-282987 treatment increased absolute cell counts of alveolar macrophages. **A.** In order to examine resident macrophage populations, cells were first gated by size to select singlets (P1), then gated for live cells using BV510 yellow viability dye. From living cells, all F4/80+ events were selected (P3) and analyzed using a dot plot, CD11b (integrin αM) VS Siglec F (resident marker). Lastly, a quadrant gate was placed in reference to the negative control, and residents were quantified in Q4 as CD11b- (PE-Cy7) F4/80+ (APC) Siglec F+ (FITC). The same gating strategy was used for Fig. 2. **B**. WT and α7nAChR-/- mice were injected with a sublethal dose of LPS. After 48h lungs were removed, digested and analyzed using flow cytometry. F4/80 positive cells were selected and examined with antibodies against CD11b and Siglec F. Resident macrophages were identified as F4/80+ Siglec F+ CD11b-. Statistical analysis was carried out using a paired t-test. *$p<0.05$.

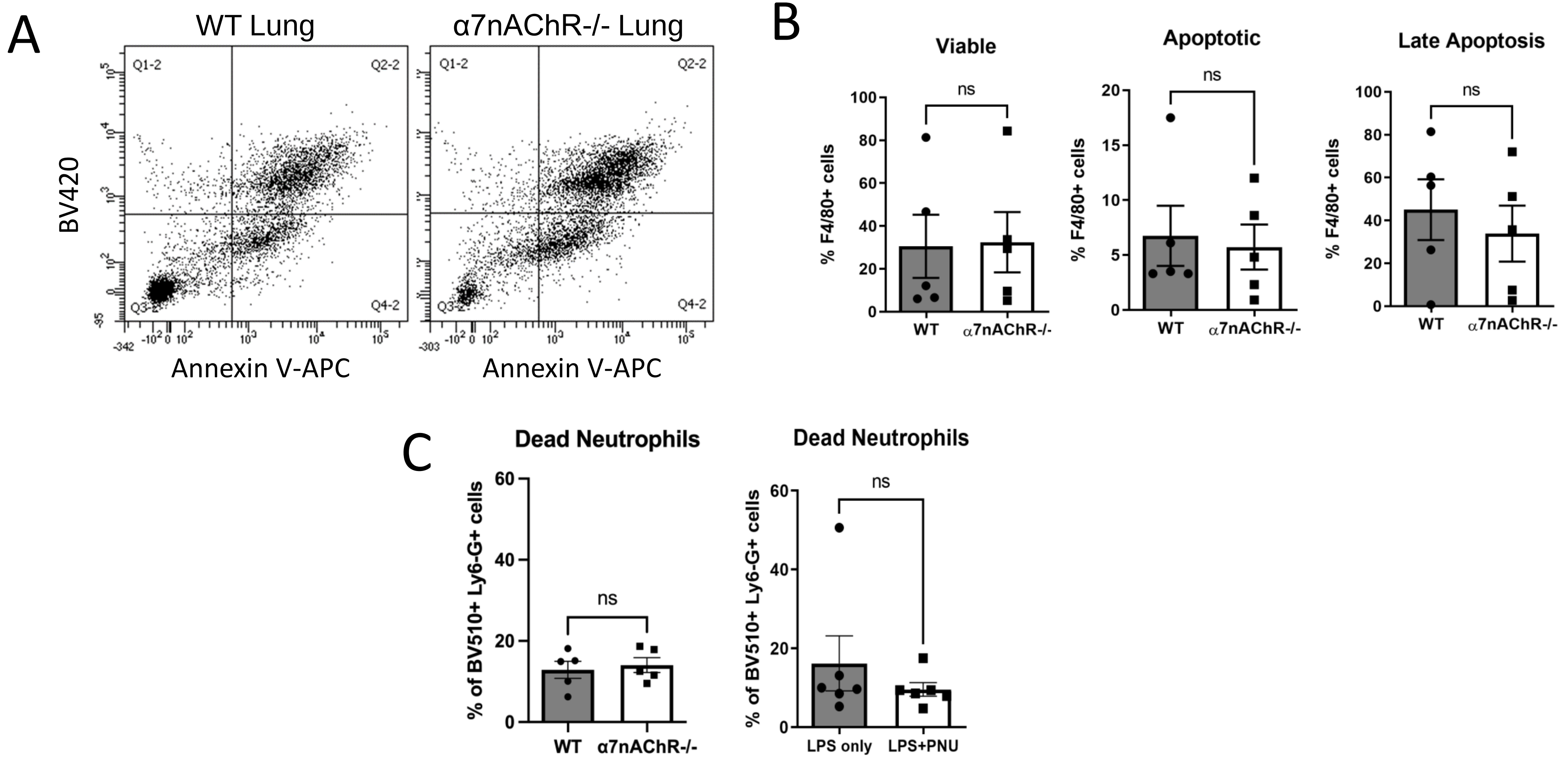

Supplemental Fig. 3. Apoptotic macrophages. A. WT and α7nAChR-/- mice were injected with a sublethal dose of LPS (n=5/group). After 48 hours the lungs were analyzed for apoptotic macrophages using flow cytometry. Using Annexin V (APC) and violet fixable viability dye (BV420), F4/80+ macrophages were analyzed for viability. Cell viability was determined by positivity for these two stains, with viable cells being those negative for both dyes (Q3-2), Annexin/BV420 double positive considered late-stage apoptosis (Q2-2), and Annexin V+ cells determined as apoptotic (Q4-2). **B.** Graphical representation of the proportion of F4/80+ cells in each viability category. Statistical analysis performed using Student's t-test. **C.** Proportions of dead neutrophils within the lungs of mice treated with or without PNU (left) and within the lungs of WT or α7nAChR-/- mice treated with LPS only (right).

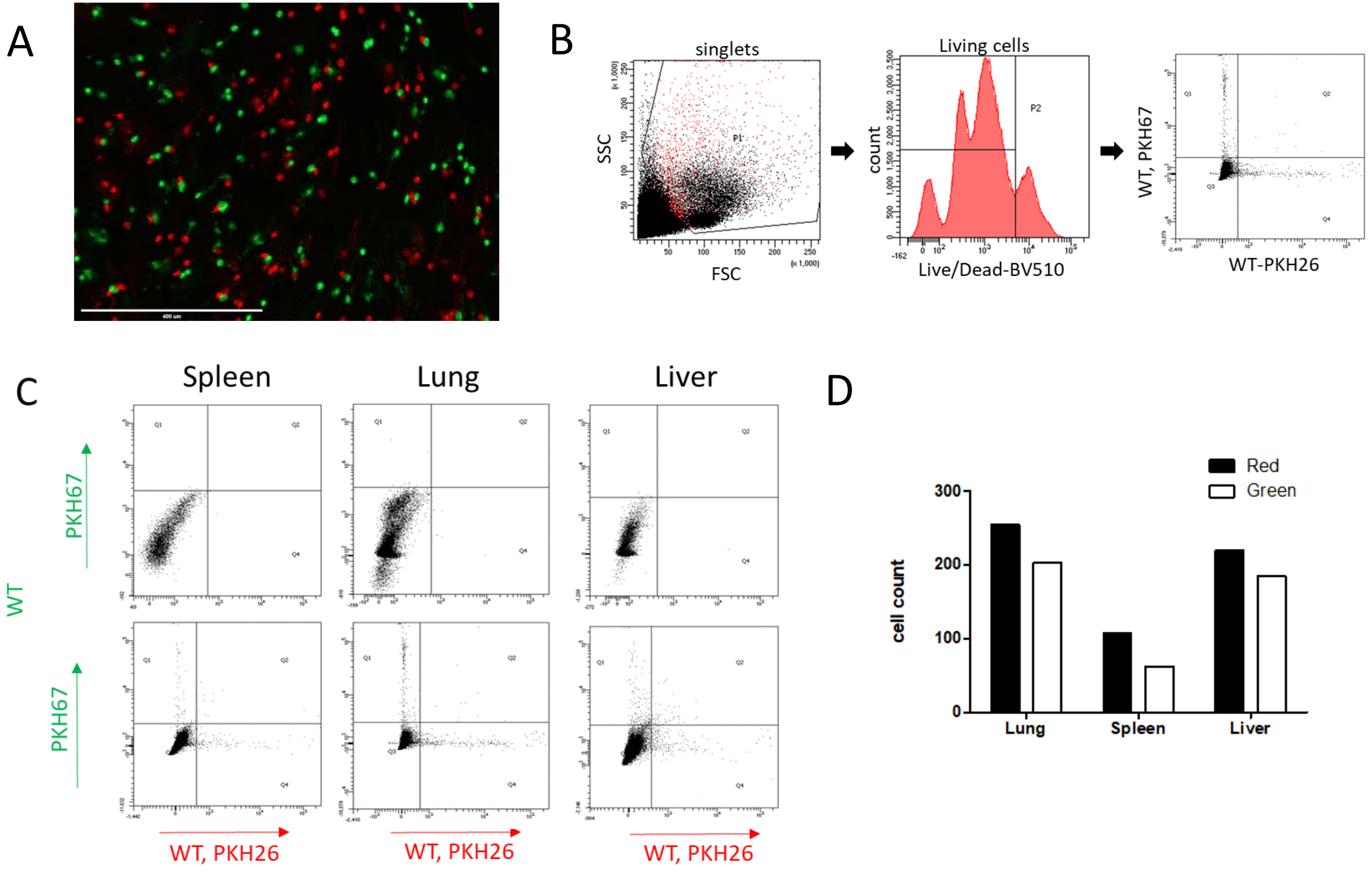

Supplemental Fig. 4 PKH dye color does not significantly affect in-vivo migration of injected monocytes. Both dye colors were used to stain WT monocytes, with experimental design and procedure otherwise identical to Fig 3. and Fig. 5. **A.** Cytospin and flow cytometry analysis checking proportion of cells in mix sample. Mix proportion was evaluated in all adoptive transfer experiments, representative image is shown. **B.** Gating strategy for the detection of labeled cells in organs. **C.** Representative flow cytometry dot plots of experimental results. **D.** Bar graphs illustrating the number of red and green WT macrophages detected in flow cytometry analysis of digested organs.

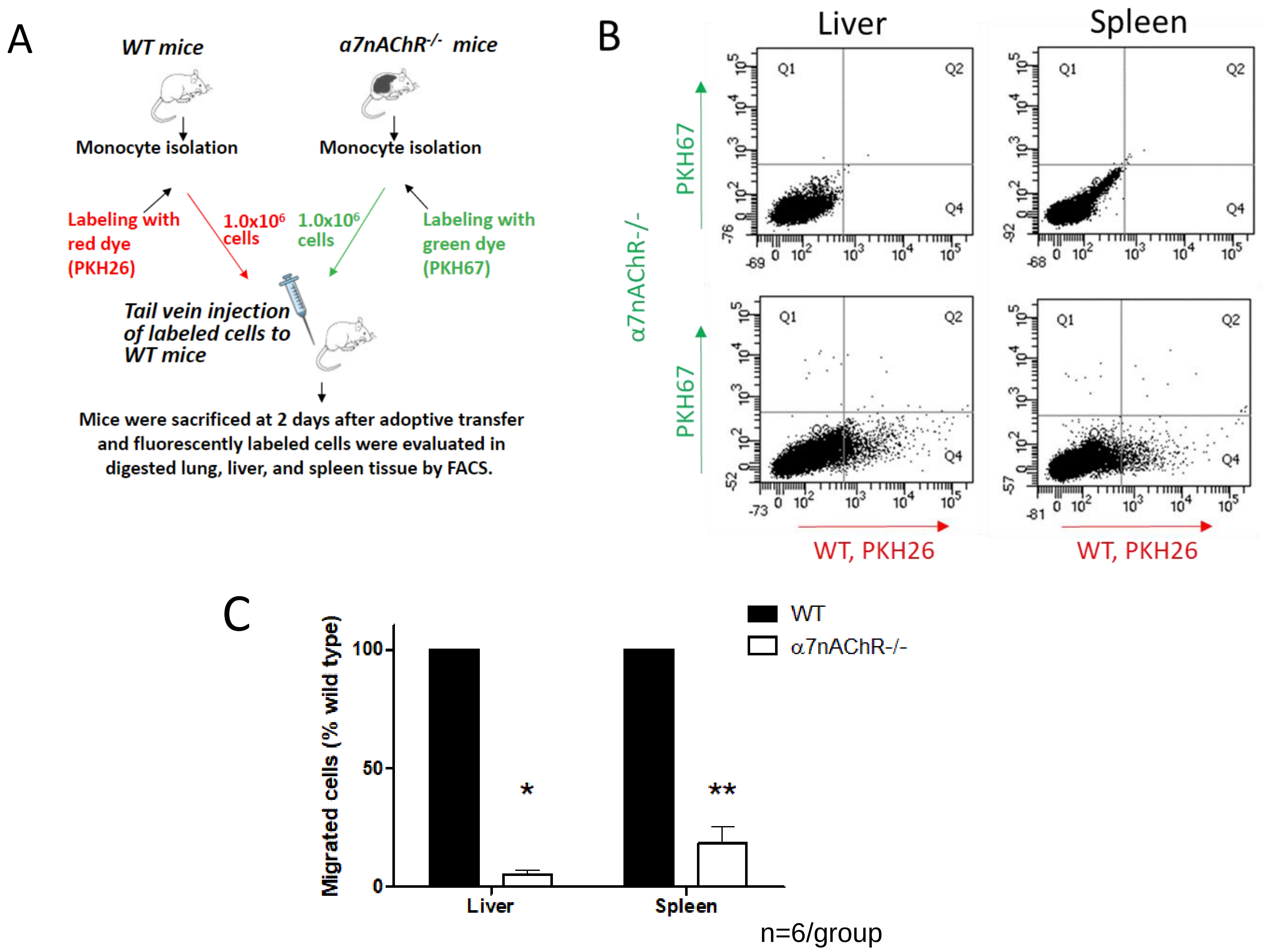

Supplemental Fig. 5. α7nAChR deficiency impedes the migration of macrophages to organs during LPS-induced endotoxemia. **A**. Schematic representation of the experimental design. Monocytes were isolated from bone marrow of WT and α7nAChR$^{-/-}$ mice via MACS. Cell were labeled with red (WT) or green (α7nAChR$^{-/-}$) fluorescent dyes, mixed in equal proportion and injected in tail vein of WT recipient mice. After 48 hours, the lung, liver and spleen were isolated, digested and analyzed using flow cytometry. **B.** Representative results of flow cytometry analysis are shown. Data was analyzed using FACSDiva software. Migrated WT macrophages (labeled with red dye) were detected in Quadrant 4; α7nAChR-/- macrophages (labeled with green dye) were detected in Quadrant 1. **C.** Bar graphs representing the amount of WT and α7nAChR-deficient macrophages detected in organs by flow cytometry. Statistical analysis was performed using Student's t-test. *, P<0.05, ** P<0.01.

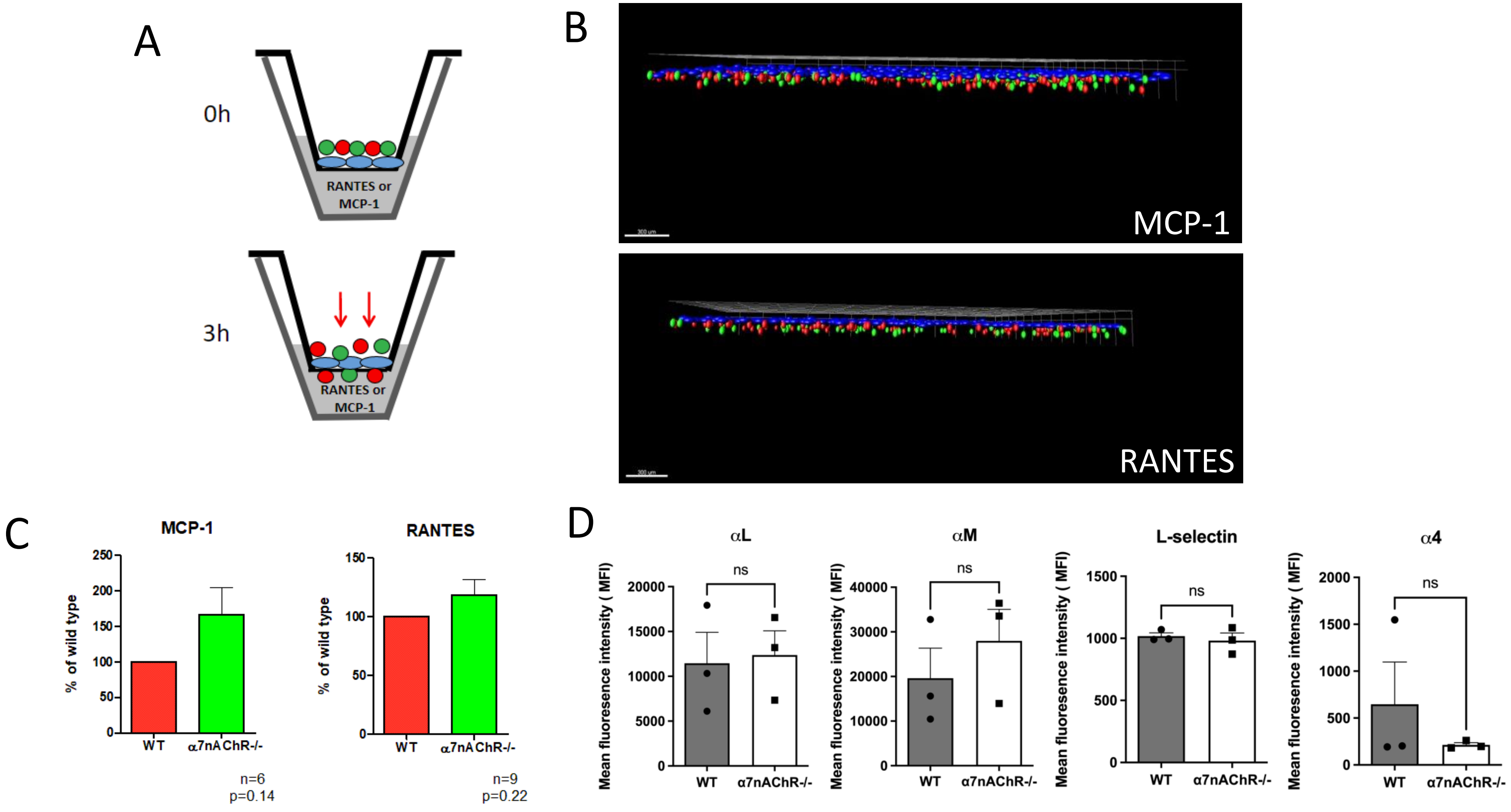

Supplemental Fig. 6. Trans-endothelial migration of monocytes along MCP-1 and RANTES gradients. **A.** Schematic representation of experimental setup within a Corning transwell insert**. B.** 2-D view of representative transwell image after 3 hours. Top, image showing transmigration in response to MCP-1 (n=6). Bottom, image showing transmigration to RANTES (n=9). **C.** Plots showing the number of monocytes migrating across the endothelial monolayer, as a percentage of WT. **D.** Plot of CD11a, CD11b, and CD62L expression on the surface of mouse peripheral blood monocytes, analyzed using flow cytometry (n=3). Statistical analysis was carried out using a student's t-test.

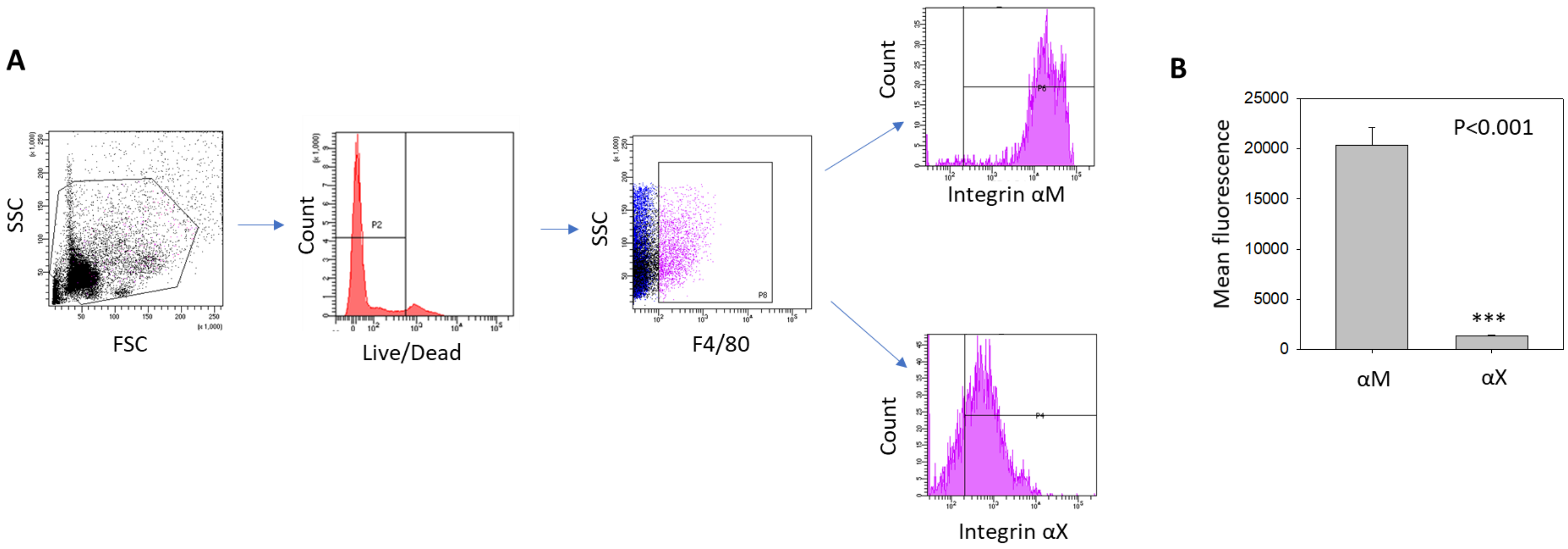

Supplemental Fig. 7. The expression of integrins αM and αX in lungs of wild type mice at 48 hours after LPS administration. A. To analyze receptor expression, cells were gated by size to select singlets from all events (P1). Next, living cells were selected by gating cells negative for yellow fixable dye, BV510 (P2). From living cells, alive cells were analyzed using a dot plot to select F4/80 –positive events (macrophage marker). The expression of integrins was detected in the macrophage-positive population. **B**. The results from 5 independent experiments were analyzed and presented as a bar graph showing the average mean fluorescence. (n=5, *** P<0.001).
